# Influence of community-level sanitation coverage and population density on environmental fecal contamination and child health in a longitudinal cohort in rural Bangladesh

**DOI:** 10.1016/j.ijheh.2022.114031

**Published:** 2022-08

**Authors:** Jesse D. Contreras, Mahfuza Islam, Andrew Mertens, Amy J. Pickering, Laura H. Kwong, Benjamin F. Arnold, Jade Benjamin-Chung, Alan E. Hubbard, Mahfuja Alam, Debashis Sen, Sharmin Islam, Mahbubur Rahman, Leanne Unicomb, Stephen P. Luby, John M. Colford, Ayse Ercumen

**Affiliations:** a Department of Forestry and Environmental Resources, North Carolina State University, Raleigh, North Carolina, 27695, United States; b Environmental Interventions Unit, Infectious Disease Division, icddr,b, Dhaka, 1212, Bangladesh; c Division of Epidemiology and Biostatistics, School of Public Health, University of California, Berkeley, Berkeley, California, 94720, United States; d Department of Civil and Environmental Engineering, University of California, Berkeley, Berkeley, California, 94720, United States; e Division of Environmental Health Sciences, School of Public Health, University of California, Berkeley, Berkeley, California, 94720, United States; f Francis I. Proctor Foundation, University of California, San Francisco, San Francisco, California, 94158, United States; g Department of Epidemiology and Population Health, Stanford University, Palo Alto, California, 94304, United States; h Infectious Diseases and Geographic Medicine, Stanford University, Stanford, California, 94305, United States

**Keywords:** Sanitation coverage, Latrine coverage, Fecal contamination, Diarrheal disease, WASH

## Abstract

**Background:**

Household-level sanitation interventions have had limited effects on child health or environmental contamination, potentially due to low community coverage. Higher community-level coverage with safely managed sanitation can reduce opportunities for disease transmission.

**Methods:**

We estimated associations between community sanitation coverage, environmental fecal contamination, and child health among 360 compounds in the control arm of the WASH Benefits trial in rural Bangladesh (NCT01590095). In each compound, we enumerated *E. coli* in environmental samples and recorded the 7-day prevalence of caregiver-reported diarrheal disease and acute respiratory infections (ARI) in children under five. We observed indicators of latrine access and quality among all neighboring compounds within 100 m of study compounds. We defined community coverage as the proportion of neighboring compounds with (1) at least one latrine, and (2) exclusively hygienic latrines (improved facility observed to safely contain feces), within both 50 m and 100 m of study compounds. We assessed effect modification by population density and season.

**Results:**

Adjusted for confounders, study compounds surrounded by 100% coverage of at least one latrine per compound within 50 m had slightly lower log_10_*E. coli* counts in stored water (Δlog = −0.13, 95% CI -0.26, −0.01), child hand rinses (Δlog = −0.13, 95% CI -0.24, −0.02), and caregiver hand rinses (Δlog = −0.16, 95% CI -0.29, −0.03) and marginally lower prevalence of diarrheal disease (prevalence ratio [PR] = 0.82, 95% CI 0.64, 1.04) and ARI (PR = 0.84, 95% CI 0.69, 1.03) compared to compounds surrounded by <100% coverage. Effects were similar but less pronounced at 100 m. At higher population densities, community latrine coverage was associated with larger reductions in *E. coli* on child and caregiver hands and prevalence of diarrheal disease. Coverage with exclusively hygienic latrines was not associated with any outcome.

**Conclusion:**

Higher community sanitation coverage was associated with reduced fecal contamination and improved child health, with stronger effects at highly local scales (50m) and at high population densities. Our findings indicate that the relationship between community sanitation coverage, environmental contamination, and child health varies by definition of coverage, distance, and population density. This work highlights significant uncertainty around how to best measure sanitation coverage and the expected health effects of increasing sanitation coverage using a specific metric. Better understanding of community-level sanitation access is needed to inform policy for implementing sanitation systems that effectively protect community health.

## 1 Introduction

Interventions to provide or promote on-site improved pit latrines have had limited effects on environmental fecal contamination (Sclar et al., 2016), diarrheal disease (Contreras and Eisenberg, 2020) and child growth (Humphrey et al., 2019; Luby et al., 2018; Null et al., 2018). One potential explanation for the null effects of many sanitation interventions is low community-level coverage achieved by most trials, whether by design or due to low intervention uptake (Pickering et al., 2019). Household-level access to an improved latrine is intended to prevent transmission of fecal-borne pathogens by separating household members from their own waste, but it does not account for transmission pathways that originate from outside the home, including environmental contamination from neighbors that do not have safely managed sanitation facilities. Humans, animals, and flies can carry fecal pathogens into the home environment from outside sources, and pathogens can infiltrate into surface- and groundwater sources from neighbors’ open defecation or unhygienic latrines, contributing to transmission independent of household-level sanitation access (Julian, 2016; Knappett et al., 2011, 2012). Increasing the proportion of households in the community with safely managed sanitation facilities hypothetically could reduce transmission by limiting opportunities for pathogen spread from outside the home, as fewer people in the community are contributing uncontained feces. However, even high community-level sanitation coverage would leave several contamination sources unaddressed, such as free-roaming domestic animals (Baker et al., 2018; Zambrano et al., 2014), exposure to untreated fecal waste through irrigation, manure application, or uncontained fecal streams (Dickin et al., 2016), contaminated produce or other food obtained outside the home (Antwi-Agyei et al., 2015; Harris et al., 2018).

Existing evidence on the relationship between community-level sanitation coverage and individual health or environmental fecal contamination is mixed but mostly suggests that increased coverage is associated with improved child health. Observational studies have found that higher levels of community-level sanitation coverage is associated with improved child growth (Fuller et al., 2016; Hammer and Spears, 2016; Harris et al., 2017; Larsen et al., 2017; Vyas et al., 2016) and reduced diarrheal disease (Andrés et al., 2017; Komarulzaman et al., 2017; Larsen et al., 2017), anemia (Kmush et al., 2021; Larsen et al., 2017), active trachoma (Garn et al., 2018), infection with *Trichuris trichiura* (Oswald et al., 2019), neonatal mortality (Kmush et al., 2021), and environmental fecal contamination (Berendes et al., 2017, 2020, 2018). A meta-analysis of observational studies found that higher community-level sanitation coverage was associated with reduced diarrheal disease (Jung et al., 2017), and a mathematical model on enteric pathogen transmission found that the entire effect of a hypothetical sanitation intervention on infection rates was due to the indirect effects of community-level coverage, rather than household-level access to sanitation (Fuller and Eisenberg, 2016).

Other observational studies have found no association between community-level sanitation coverage and diarrheal disease (Harris et al., 2017), hookworm infection (Oswald et al., 2019), and environmental fecal contamination (Huda et al., 2019; Odagiri et al., 2016). A study on the spillover effects from a combined water, sanitation, and hygiene (WASH) intervention among nearby neighbors of intervention recipients found reduced *E. coli* in stored drinking water from tubewells but no difference in fecal contamination through other environmental pathways, helminth infections, diarrheal disease, or respiratory illness (Benjamin-Chung et al., 2018). A meta-analysis of intervention trials found no clear association between sanitation coverage and diarrheal disease, except among sewerage interventions (Contreras and Eisenberg, 2020; Wolf et al., 2018).

Most of the research on community-level sanitation measured coverage through surveys conducted among a subset of residents over large sampling areas. Few studies have comprehensively measured sanitation coverage for all households within the sampling area to capture both latrine presence and quality (Fuller et al., 2016; Harris et al., 2017; Huda et al., 2019), and no studies have assessed both child health and fecal contamination to assess if any health benefits of coverage are causally supported by reductions in contamination and to investigate which environmental pathways are most influenced by community-level sanitation coverage. The objective of this analysis was to estimate associations between community-level sanitation coverage within a proximate (50–100 m) radius of study participants, environmental fecal contamination along multiple pathways, and child health in a longitudinal study nested within a randomized controlled trial in rural Bangladesh (Luby et al., 2018). In addition, we analyzed population density and season as potential modifiers of the relationship between community-level sanitation coverage and each outcome.

## 2 Materials and methods

### 2.1 Study design

This study was conducted in a longitudinal cohort nested within the control arm of the WASH Benefits randomized controlled trial in rural Bangladesh (Luby et al., 2018). The trial enrolled multifamily compounds that included a pregnant woman in her first or second trimester. The household in which the pregnant woman lived was the target household. Enrolled compounds were grouped into clusters of 6–8 spatially contiguous compounds, and clusters were randomly assigned to one of six WASH intervention arms or into the control arm. We randomly selected 360 (of 696) compounds from the sanitation arm and 360 (of 1,382) compounds from the control arm of the parent trial to participate in a longitudinal substudy focused on environmental contamination. The present analysis includes data from the control arm of the substudy to capture sanitation conditions unaltered by the intervention. Participants provided written informed consent in Bengali. The study protocol was approved by human subjects committees at the icddr,b (PR-11063), University of California, Berkeley (2011–09−3652), and Stanford University (25863).

### 2.2 Data collection

Compounds participating in the substudy were visited eight times over 30 months. GPS coordinates were recorded at the entrance of the target household during the first visit. Samples were collected during each visit from various locations within the compound environment representing potential pathways of contamination from fecal sources. Stored drinking water samples and hand rinses from children and caregivers were collected at each visit. Samples of soil from the courtyard at the entrance of the target household and stored food for young children were collected during the third and fourth visits only. Samples were processed at the local field lab of the icddr,b on the same day as collection with IDEXX Quanti-Tray/2000 to enumerate the most probable number (MPN) of *E. coli*. Methods for sample collection and analysis have been detailed elsewhere (Contreras et al., 2021; Ercumen et al., 2018).

At each visit, field staff administered a survey that included caregiver-reported symptoms of diarrheal disease and acute respiratory infection (ARI) for all children under five years of age in the compound, sanitation behaviors (e.g., latrine use, open defecation by children), and presence and number of domestic animals (cattle, goats, sheep, pigs, poultry, dogs, and cats). Field staff also completed spot check observations of sanitary conditions, such as the type and hygienic condition of each latrine in the compound.

During the second visit, a community survey was conducted to measure population density and sanitation coverage within a 100 m radius of study compounds. Field staff identified all compounds within this range by walking 300 steps (approximately 100 m) in each direction away from the study compound. At each compound within this radius, they recorded the total number of people who lived in the compound, the number of latrines in the compound, and GPS coordinates at the entrance of the compound and at each latrine. They observed and recorded the type and hygienic condition of each latrine, including where the latrine flushed to and whether feces were fully contained within a pit or septic tank.

### 2.3 Statistical methods

#### 2.3.1 Outcome variables and parameters of interest

The primary outcomes of this study were i) counts of *E. coli* in environmental samples (stored drinking water, child hand rinse, mother hand rinse, soil, and child food), ii) diarrheal disease in children under five, and iii) ARI in children under five. *E. coli* counts were analyzed as a continuous variable representing the log_10_-transformed MPN of bacteria per unit of sample (per 100 mL of stored drinking water, per two hands for rinses, or per one dry gram of food/soil) and were analyzed separately by sample type. Samples without detectable levels of *E. coli* were assigned a value equal to half the lower detection limit. Diarrheal disease and ARI were operationalized as binary variables based on caregiver-reported symptoms for a seven-day recall period. Diarrheal disease was defined as passing three or more loose or watery stools or at least one stool with blood. ARI was defined as persistent cough, panting, wheezing, or difficulty breathing. Both child health outcomes were analyzed at the child level with observations pooled across sampling rounds, such that each child under five provided up to eight data points for each outcome. The parameters of interest for this analysis were i) log_10_ reductions in *E. coli* for each sample type and ii) prevalence ratios and prevalence differences for diarrheal disease and ARI, associated with different levels of community sanitation coverage.

#### 2.3.2 Exposure variables

We aimed to capture multiple aspects of community-level sanitation coverage by using two exposure definitions (any latrine coverage and hygienic latrine coverage), operationalizing these exposures in both binary and continuous forms, and quantifying them within two different radii around study compounds (50 m and 100 m).

We defined “any latrine coverage” as the proportion of neighboring compounds with at least one latrine within the specified radius around each study compound. This definition identifies compounds that have no latrine access, while ignoring latrine quality within compounds with at least one latrine. Although compounds without their own latrines may use latrines in public locations (e.g. mosques) or in other compounds, we assume that lack of latrine access within the compound might indicate some degree of open defecation. We operationalized any latrine coverage as a binary indicator variable for whether 100% of compounds within the specified radius of the study compound had at least one latrine (100% vs. <100% coverage). This comparison reflects the impact of complete coverage with at least one latrine per compound and assumes that even one compound relying on open defecation can impact community contamination and disease transmission. We also aimed to assess the effect of any latrine coverage as a continuous variable (0%–100%). However, for most study compounds, 80–100% of neighboring compounds had at least one latrine. Therefore, we did not analyze a continuous form of any latrine coverage.

We defined “hygienic latrine coverage” as the proportion of neighboring compounds with exclusively hygienic latrines (i.e., at least one latrine in the compound and all latrines in the compound were hygienic) within the specified radius of each study compound. We defined “hygienic latrine” as an improved facility that does not drain into the environment and where feces are fully contained within the pit or septic tank, based on observations by field staff (UNICEF et al., 2019). This definition captures the role of latrine quality but does not differentiate between defecation in a non-hygienic latrine and open defecation. We operationalized hygienic latrine coverage as a binary indicator variable for whether 100% of compounds within the specified radius of the study compound had exclusively hygienic latrines (100% vs. <100% coverage). This comparison reflects the impact of complete coverage with high-quality latrines and assumes that even one non-hygienic latrine can lead to environmental contamination and pathogen spread. We also analyzed hygienic latrine coverage as a continuous variable (0%–100%) to assess the incremental effect of increasing coverage.

For both of these exposure definitions, we chose a maximum radius of 100 m from study compounds to reflect the upper range of the distance fecal pathogens have been shown to travel in the subsurface from pit latrines (Graham and Polizzotto, 2013). However, it is possible that neighbors’ sanitation coverage impacts target households over shorter distances through other pathways, such as direct contact between household residents or animals. To assess the role of distance in our analysis, we used GPS data to quantify each exposure within 50 m and 100 m of study compounds.

#### 2.3.3 Estimation strategy

Parameters were estimated through generalized linear models, with robust standard errors to account for data clustering and repeated measures. Log_10_ *E. coli* differences and prevalence differences were estimated using a Gaussian distribution (link = identity), and disease prevalence ratios were estimated using a binomial distribution (link = log). For binomial models that did not converge, a modified Poisson (link = log) distribution was used instead. For hygienic latrine coverage in its continuous form, we divided percent coverage by 10 so that model estimates reflect a 10 percentage point increase in coverage. We pre-specified smoothing spline regression with three knots; however, we found no differences between spline segments so instead modeled continuous hygienic latrine coverage without spline terms. We conducted both unadjusted and adjusted analyses. We pre-specified a list of potential confounders for each outcome based on plausible causal pathways and included all variables that were associated with the outcome in bivariate regression models (p < 0.20) as covariates in adjusted models. Potential covariates included indicators of socioeconomic status, hygienic latrine access, open defecation of young children, and the number of domestic animals by type in the study compound. The full list of potential covariates and included covariates for each outcome can be found in Supplementary Materials (Table S1).

We assessed effect modification by population density (defined as the number of people living within the specified radius of the study compound) and season (monsoon vs. dry season). Effect modification by season was not included in our pre-specified analysis plan; we added this analysis post hoc because we found stronger intervention effects from sanitation improvements in the parent trial during the monsoon season. We operationalized population density both as a continuous variable and in tertiles. We defined the monsoon season by year using daily rainfall data recorded by the Bangladesh Meteorological Department at three weather stations nearest the study region between 2014 and 2016 (Zaman, 2018). We calculated five-day rolling averages of daily rainfall at each station and defined the monsoon season for each year as the period between the first and last days with a five-day rolling average rainfall of 10 mm or greater at any station. Monsoon seasons were April 2-September 27, 2014, March 31-September 25, 2015, and March 30-October 30, 2016. We conducted subgroup analyses for each exposure-outcome relationship within each tertile of population density and within each season to qualitatively assess effect modification. We added an interaction term between each exposure variable and the potential effect-modifying variable (continuous population density or binary season) to adjusted models. We interpreted a p-value <0.2 on the interaction term as quantitative evidence of significant effect modification (Thiese et al., 2016). Soil and food samples were excluded from effect modification analyses due to sample size.

In primary models, we pooled outcomes measured across all eight sampling rounds. This analysis assumes that the community-level sanitation variables did not change significantly over time. As a sensitivity analysis, we restricted models of exposures within 100 m to outcomes measured during the second data collection round, when community-level sanitation variables were measured. Soil and food samples were not collected during the second round and were not included in sensitivity analyses. In addition, we considered the role of spatial autocorrelation of outcomes in our analysis. We found evidence of spatial clustering of outcomes before analysis (results available in our pre-specified analysis plan: https://osf.io/6u7cn/) and therefore assessed spatial autocorrelation in residual error values for each study compound from adjusted continuous models using Moran's I. We found no evidence of residual spatial autocorrelation for any outcome (Table S2).

## 3 Results

Over eight sampling rounds, participants from 360 study compounds completed 2,679 data collection visits. We collected a total of 2,317 stored water samples, 2,621 child hand rinses, 2,656 caregiver hand rinses, 385 soil samples, and 273 stored food samples. Health data were reported for 867 individual children under five for a total of 4,712 child observations over eight rounds. Mean log_10_-transformed *E. coli* counts were 1.02 (standard deviation [sd] = 1.05) in stored water, 1.50 (sd = 1.01) in child hand rinses, 1.48 (sd = 1.02) in caregiver hand rinses, 5.21 (sd = 1.07) in soil, and 1.77 (sd = 1.36) in stored food. The overall prevalence of diarrheal disease and acute respiratory infection among children under five was 14.5% and 22.7%, respectively.

### 3.1 Community-level sanitation coverage

Of the 360 study compounds, 342 had neighboring compounds within 50 m (median n neighbors = 4, range 1–23) and 358 had neighbors within 100 m (median n neighbors = 10, range 1–43) who were captured by our community survey and included in the analysis of any latrine coverage. One study compound was missing data on the hygienic status of neighboring latrines, resulting in 341 and 357 study compounds for analysis of exclusively hygienic latrines within 50 and 100 m, respectively.

**Fig. 1:**
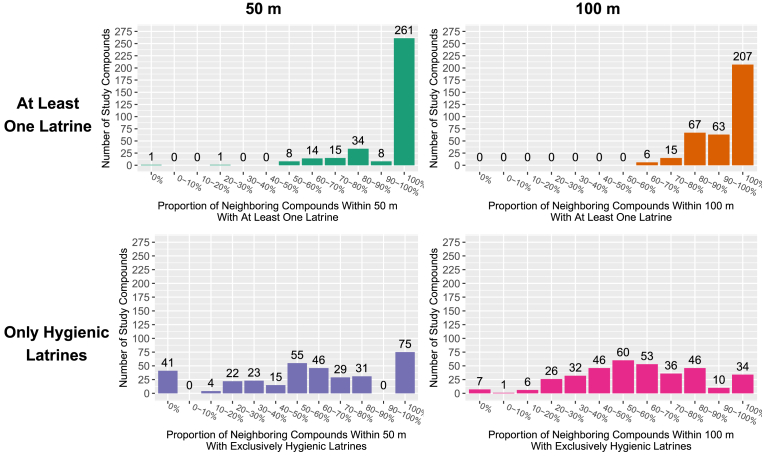
Distribution of community-level any latrine coverage (the proportion of compounds within range with at least one latrine; top row) and community-level hygienic latrine coverage (the proportion of compounds within range with only hygienic latrines; bottom row). Distributions plotted within a radius of 50 m (left column) and 100 m (right column) around study compounds.

Among study compounds with neighbors present within the specified range, for 261 (76%) compounds, 100% of neighboring compounds within 50 m had at least one latrine and for 207 (58%) compounds, 100% of neighboring compounds within 100 m had at least one latrine (Table 1; Fig. 1). Within the 50 m radius, for 75 compounds (22%), 100% of neighbors had exclusively hygienic latrines (Fig. 1). Within the 100 m radius, for 34 compounds (10%), 100% of neighbors had exclusively hygienic latrines (Fig. 1; Table 1). Overall, enrolled compounds with 100% community-level sanitation coverage were more likely to be food secure, wealthier, use a hygienic latrine themselves, and report no open defecation for children aged 3–8 compared to those with <100% coverage (Table 1).

**Table 1:** Baseline characteristics by sanitation coverage within 50 m and 100 m of study compounds.

	50 m Radius Around Study Compounds				100 m Radius Around Study Compounds			
	Proportion of Compounds with At Least One Latrine		Proportion of Compounds with Only Hygienic Latrines		Proportion of Compounds with At Least One Latrine		Proportion of Compounds with Only Hygienic Latrines	
	<100% coverage n = 81	100% coverage n = 261	<100% coverage n = 266	100% coverage n = 75	<100% coverage n = 151	100% coverage n = 207	<100% coverage n = 323	100% coverage n = 34
***Measured at Baseline of Parent Trial***								
Maternal years of education, median (sd)	5 (3.5)	7 (3.4)	6 (3.4)	7 (3.4)	5 (3.3)	7 (3.4)	6 (3.4)	8 (3.3)
Mother's age in years, median (sd)	23 (4.5)	23 (5.1)	23 (4.8)	23 (5.5)	23 (5.0)	23 (5.0)	23 (4.8)	20.5 (6.2)
Food insecurity, n (%)								
*Food secure*	54 (67)	183 (70)	181 (68)	55 (73)	99 (66)	148 (71)	219 (68)	27 (79)
*Mildly food insecure*	7 (9)	20 (8)	21 (8)	6 (8)	14 (9)	15 (7)	27 (8)	2 (6)
*Moderately food insecure*	19 (23)	48 (18)	54 (20)	13 (17)	34 (23)	37 (18)	66 (20)	5 (15)
*Severely food insecure*	1 (1)	10 (4)	10 (4)	1 (1)	4 (3)	7 (3)	11 (3)	0 (0)
Wealth, n (%)								
*Quartile 1 (Least Wealth)*	24 (30)	51 (20)	64 (24)	11 (15)	38 (25)	40 (19)	75 (23)	3 (9)
*Quartile 2*	24 (30)	67 (26)	73 (27)	18 (24)	43 (28)	52 (25)	89 (28)	6 (18)
*Quartile 3*	14 (17)	66 (25)	63 (24)	17 (23)	33 (22)	51 (25)	76 (24)	7 (21)
*Quartile 4 (Most Wealth)*	19 (23)	77 (30)	66 (25)	29 (39)	37 (25)	64 (31)	83 (26)	18 (53)
Number of children <18 in the target household, median (sd)	1 (1.2)	1 (1.2)	1 (1.1)	1 (1.2)	1 (1.2)	1 (1.2)	1 (1.2)	1 (1.4)
Number of individuals living in the target household, median (sd)	4 (2.5)	4 (2.1)	4 (2.3)	4 (1.9)	4 (2.2)	4 (2.2)	4 (2.2)	4.5 (2.0)
Distance in minutes to target household's primary drinking water source, median (sd)	0 (1.3)	0 (1.2)	0 (1.2)	0 (1.2)	0 (1.5)	0 (1.1)	0 (1.3)	0 (0.9)
Improved roof, n (%)	81 (100)	257 (98)	263 (99)	74 (99)	150 (99)	203 (98)	318 (98)	34 (100)
Improved floor, n (%)	2 (2)	39 (15)	24 (9)	17 (23)	14 (9)	28 (14)	32 (10)	10 (29)
Improved walls, n (%)	59 (73)	166 (64)	185 (70)	39 (52)	102 (68)	132 (64)	217 (67)	17 (50)
***Measured at Baseline of Environmental Substudy***								
Primary latrine used by target household is hygienic, n (%)	46 (61)	188 (75)	173 (68)	61 (85)	94 (67)	150 (74)	213 (69)	30 (88)
Open defecation by children <3, n (%)								
*Daily*	70 (86)	208 (80)	223 (84)	54 (72)	125 (83)	164 (79)	268 (83)	21 (62)
*Occasionally*	9 (11)	49 (19)	39 (15)	19 (25)	22 (15)	40 (19)	50 (15)	11 (32)
*Never*	2 (2)	4 (2)	4 (2)	2 (3)	4 (3)	3 (1)	5 (2)	2 (6)
Open defecation by children 3–8, n (%)								
*Daily*	23 (48)	41 (31)	58 (40)	6 (17)	34 (41)	32 (31)	65 (38)	1 (7)
*Occasionally*	6 (12)	27 (20)	23 (16)	9 (26)	14 (17)	19 (18)	31 (18)	2 (14)
*Never*	19 (40)	65 (49)	64 (44)	20 (57)	34 (41)	52 (50)	75 (44)	11 (79)
Number of cattle, n (%)								
*None*	24 (30)	78 (30)	82 (31)	19 (25)	44 (29)	63 (30)	94 (29)	12 (35)
*Tertile 1 (1–2)*	25 (31)	84 (32)	76 (29)	33 (44)	54 (36)	59 (29)	102 (32)	11 (32)
*Tertile 2 (3–4)*	13 (16)	51 (20)	53 (20)	11 (15)	22 (15)	45 (22)	60 (19)	7 (21)
*Tertile 3 (5–57)*	19 (23)	48 (18)	55 (21)	12 (16)	31 (21)	40 (19)	67 (21)	4 (12)
Number of poultry, n (%)								
*None*	9 (11)	23 (9)	26 (10)	6 (8)	14 (9)	19 (9)	27 (8)	5 (15)
*Tertile 1 (1–10)*	32 (40)	67 (26)	73 (27)	25 (33)	46 (30)	56 (27)	89 (28)	13 (38)
*Tertile 2 (11–21)*	20 (25)	84 (32)	82 (31)	22 (29)	46 (30)	64 (31)	100 (31)	10 (29)
*Tertile 3 (22–132)*	20 (25)	87 (33)	85 (32)	22 (29)	45 (30)	68 (33)	107 (33)	6 (18)
Number of goats and sheep, n (%)								
*None*	46 (57)	163 (62)	164 (62)	45 (60)	94 (62)	123 (59)	194 (60)	22 (65)
*Tertile 1 (1–2)*	20 (25)	59 (23)	58 (22)	20 (27)	30 (20)	54 (26)	77 (24)	7 (21)
*Tertile 2 (3)*	6 (7)	15 (6)	16 (6)	5 (7)	10 (7)	11 (5)	18 (6)	3 (9)
*Tertile 3 (4–20)*	9 (11)	24 (9)	28 (11)	5 (7)	17 (11)	19 (9)	34 (11)	2 (6)
Number of other animals, n (%)								
*None*	75 (93)	220 (84)	229 (86)	65 (87)	135 (89)	172 (83)	278 (86)	28 (82)
*Tertile 1 (1)*	2 (2)	20 (8)	18 (7)	4 (5)	9 (6)	13 (6)	21 (7)	1 (3)
*Tertile 2 (2–3)*	2 (2)	10 (4)	9 (3)	3 (4)	4 (3)	11 (5)	12 (4)	3 (9)
*Tertile 3 (4–12)*	2 (2)	11 (4)	10 (4)	3 (4)	3 (2)	11 (5)	12 (4)	2 (6)

**Fig. 2:**
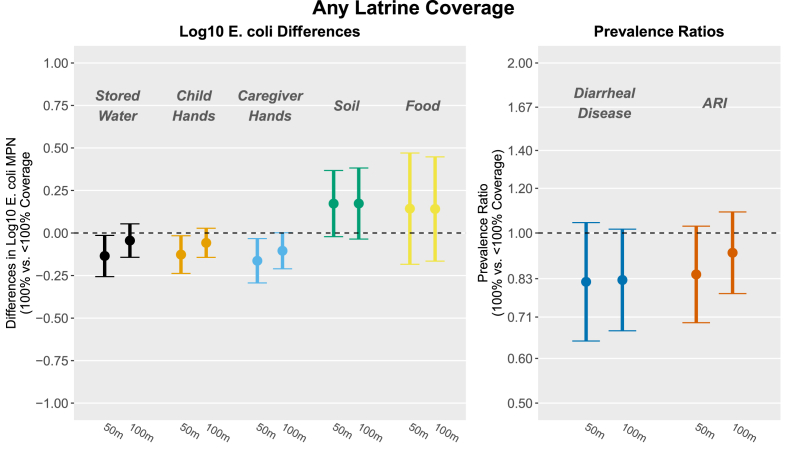
Associations between study outcomes and community-level any latrine coverage (the proportion of compounds within range with at least one latrine) within 50 m (left within each outcome) and 100 m (right) of study compounds, modeled as binary variables. Estimates reflect log_10_*E. coli* differences and diarrhea and ARI prevalence ratios comparing compounds surrounded by 100% vs. <100% coverage. All models were adjusted for relevant covariates (Table S1) and include robust standard errors.

In adjusted analyses, compounds surrounded by 100% community-level “any latrine coverage” (i.e., all compounds within range having at least one latrine) within 50 m had slightly lower log_10_ *E.coli* counts in stored water (Fig. 2; Table S3; Δlog = −0.13, 95% CI -0.26, −0.01), child hand rinses (Δlog = −0.13, 95% CI -0.24, −0.02), and caregiver hand rinses (Δlog = −0.16, 95% CI -0.29, −0.03) and marginally lower prevalence of diarrheal disease (PR = 0.82, 95% CI 0.64, 1.04) and ARI (PR = 0.84, 95% CI 0.69, 1.03) compared to compounds surrounded by <100% coverage. At 100 m, 100% any latrine coverage was marginally associated with reduced log_10_ *E. coli* counts in caregiver hand rinses (Fig. 2; Table S3; Δlog = −0.10, 95% CI -0.21, 0.00) and reduced diarrheal disease (PR = 0.83, 95% CI 0.67, 1.02).

**Fig. 3:**
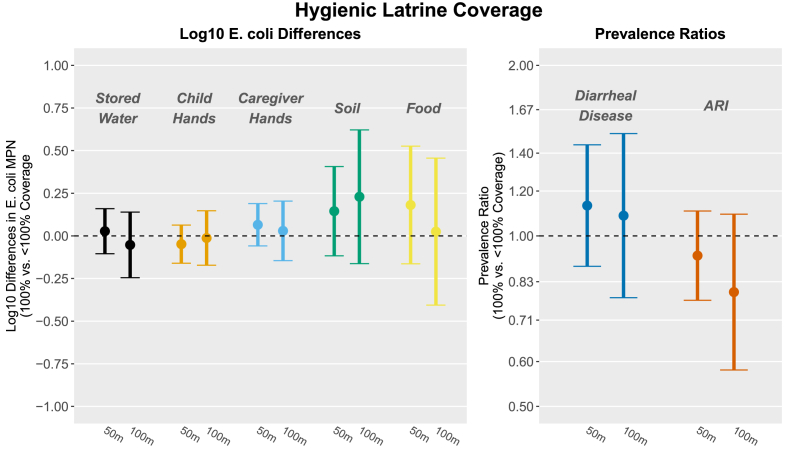
Associations between study outcomes and community-level hygienic latrine coverage (the proportion of compounds within range with only hygienic latrines) within 50 m (left within each outcome) and 100 m (right) of study compounds, modeled as binary variables. Estimates reflect log_10_*E. coli* differences and diarrhea and ARI prevalence ratios comparing compounds surrounded by 100% vs. <100% coverage. All models were adjusted for relevant covariates (Table S1) and include robust standard errors.

There were no associations between community-level “hygienic latrine coverage” (the proportion of compounds within range with only hygienic latrines) and any outcome after adjustment, including both binary and continuous forms of the exposure within a range of 50 m or 100 m (Fig. 3; Table 2; Table S4). There were marginally significant associations between hygienic latrine coverage in continuous form and lower *E. coli* counts in stored water at both 50 m and 100 m, but the magnitude of the associations was small (0.01 and 0.02 log_10_ reductions, respectively) (Table 2). Results were not significantly different for any exposure using outcome data from the second sampling round only, when the community coverage variables were measured (Tables S5–S7).

**Table 2:** Results for continuous exposure definition. Adjusted and unadjusted associations between study outcomes and community-level hygienic latrine coverage (the proportion of compounds within range with only hygienic latrines), modeled as a continuous exposure. Estimates reflect changes in study outcomes associated with a 10 percentage point increase in hygienic latrine coverage. Exposures were modeled for two different radii (50 m and 100 m) around study compounds. All models include robust standard errors.

	50 m				100 m			
	Unadjusted		Adjusted^a^		Unadjusted		Adjusted^a^	
	*n*	Estimate	*n*	Estimate	*n*	Estimate	*n*	Estimate
***Log10 E. coli MPN Differences***								
Stored Water	2204	−0.02 (−0.04, 0.00)*	2123	−0.01 (−0.03, 0.00)	2299	−0.04 (−0.06, −0.02)*	2214	−0.02 (−0.04, 0.00)
Child Hand Rinses	2488	−0.01 (−0.03, 0.00)	2443	0.00 (−0.02, 0.01)	2598	−0.02 (−0.04, 0.00)	2550	−0.01 (−0.03, 0.01)
Caregiver Hand Rinses	2522	0.00 (−0.02, 0.02)	2486	0.01 (−0.01, 0.03)	2633	−0.02 (−0.04, 0.01)	2594	0.00 (−0.02, 0.02)
Soil	364	−0.03 (−0.07, 0.00)*	359	0.02 (−0.01, 0.06)	382	−0.07 (−0.12, −0.03)*	377	0.01 (−0.04, 0.06)
Food	265	0.02 (−0.03, 0.07)	265	0.02 (−0.03, 0.07)	273	0.02 (−0.04, 0.07)	273	0.00 (−0.06, 0.06)
***Prevalence Ratios***								
Diarrheal Disease	4454	1.00 (0.96, 1.03)	4419	1.00 (0.97, 1.03)	4652	0.99 (0.94, 1.04)	4617	1.00 (0.96, 1.05)
Acute Respiratory Infection (ARI)	4457	0.99 (0.96, 1.01)	4422	1.00 (0.97, 1.02)	4655	0.99 (0.96, 1.02)	4620	1.01 (0.97, 1.04)
***Prevalence Differences***								
Diarrheal Disease	4454	0.00 (−0.01, 0.00)	4419	0.00 (0.00, 0.00)	4652	0.00 (−0.01, 0.01)	4617	0.00 (−0.01, 0.01)
Acute Respiratory Infection (ARI)	4457	0.00 (−0.01, 0.00)	4422	0.00 (−0.01, 0.01)	4655	0.00 (−0.01, 0.01)	4620	0.00 (−0.01, 0.01)

*Statistically significant at the 0.05 level.

a See Table S1 for full list of potential and selected covariates by outcome.

### 3.2 Effect modification

**Fig. 4:**
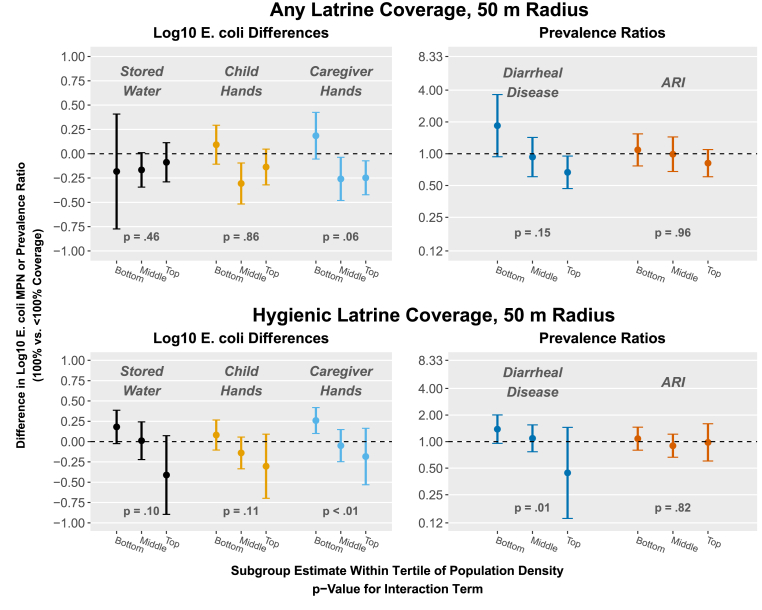
Effect modification by population density on community-level any latrine coverage (the proportion of compounds within range with at least one latrine) and community hygienic latrine coverage (the proportion of compounds within range with only hygienic latrines) within 50 m of study compounds and study outcomes. Exposures were modeled as binary variables (100% vs. <100% coverage). Plots show subgroup estimates within tertiles of population density. P-values are for the interaction term between continuous population density and the exposure. All models are adjusted and include robust standard errors.

The median number of people living within 50 m of study compounds was 33 (tertiles = 0–21, 22–45, 46–159). The median number of people living within 100 m was 82 (tertiles = 0–62, 63–111, 112–354). Within 50 m of study compounds, population density modified the association between any latrine coverage and *E. coli* on caregiver hands (interaction p-value = 0.06) and diarrheal disease (interaction p-value = 0.15) (Fig. 4; Table S8). In the middle and highest population density tertiles, compounds surrounded by 100% any latrine coverage had approximately 0.25-log_10_ lower *E. coli* counts on child and caregiver hands than those surrounded by <100% coverage; there was no association in the lowest tertile. Similarly, in the highest tertile of population density, children in compounds surrounded by 100% any latrine coverage had 33% lower prevalence of diarrhea (PR = 0.67, 95% CI 0.47, 0.95). Also within 50 m, population density modified the association between 100% hygienic latrine coverage and all outcomes other than ARI (interaction p-values<0.2) (Fig. 4; Table S9). Qualitatively, being surrounded by 100% hygienic latrine coverage was associated with progressively larger reductions in *E. coli* counts and diarrhea prevalence as population density increased but most subgroup estimates included the null. Results were generally similar using the 100 m radius (Fig. S1; Tables S10–S11) and for the continuous form of hygienic latrine coverage (Tables S12–S13).

**Fig. 5:**
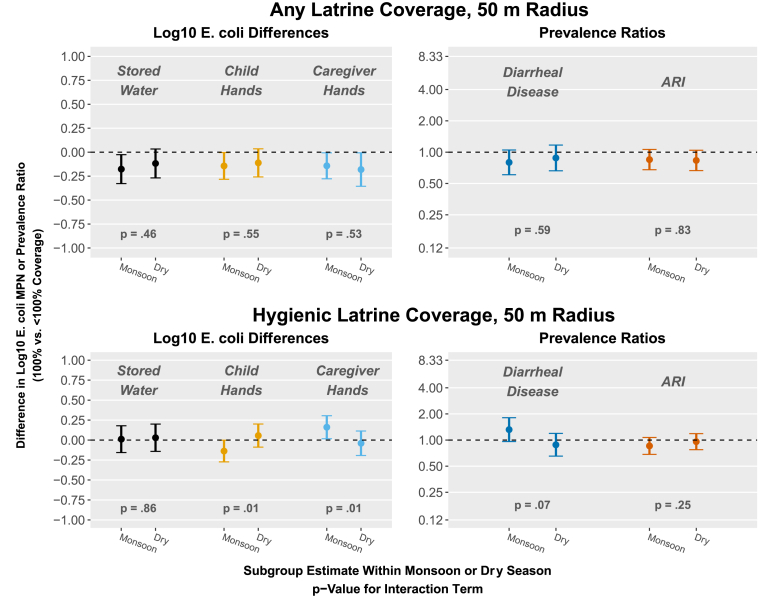
Effect modification by season (monsoon vs. dry) on community-level any latrine coverage (the proportion of compounds within range with at least one latrine) and community hygienic latrine coverage (the proportion of compounds within range with only hygienic latrines) within 50 m of study compounds and study outcomes. Exposures were modeled as binary variables (100% vs. <100% coverage). Plots show subgroup estimates within each season. P-values are for the interaction term between season and the exposure. All models are adjusted and include robust standard errors.

Season did not modify any outcome association with having 100% any latrine coverage at 50 m or 100 m (Fig. 5; Table S14; Fig. S2; Table S16). Within 50 m of study compounds, season modified the association between having 100% hygienic latrine coverage and log_10_ *E. coli* counts on child hands and caregiver hands (in opposite directions) and the prevalence of diarrheal disease (Fig. 5; Table S15). During monsoon seasons, being surrounded by 100% hygienic latrine coverage within 50 m was associated with reduced *E. coli* contamination on child hands (Δlog = −0.14, 95% CI -0.27, 0.00) and increased contamination on caregiver hands (Δlog = 0.16, 95% CI 0.02, 0.30), but both magnitudes were small. Being surrounded by 100% hygienic latrine coverage was marginally associated with an increase in the prevalence of diarrheal disease (PR = 1.32, 95% CI 0.96, 1.81) in the monsoon season. Results were similar but weaker for the 100 m radius and for continuous hygienic latrine coverage (Fig. S2; Tables S17–S19).

## 4 Discussion

We found that 100% community-level coverage, with all neighboring compounds within 50 m of study compounds having at least one latrine, was associated with slightly lower counts of *E. coli* in stored drinking water and caregiver and child hand rinses (about 0.15-log_10_ lower MPN) and marginally associated with reduced prevalence of diarrheal disease and ARI (about 17% relative reduction) among study compounds. These associations were attenuated when we evaluated coverage within 100 m of study compounds. Community-level coverage with hygienic latrines was not associated with *E. coli* counts, diarrhea or ARI using either radius. Associations between coverage and *E. coli* on child or caregiver hands and diarrheal disease were consistently modified by population density, with coverage more strongly associated with outcomes for compounds in areas with higher population density. We found no strong evidence of effect modification by monsoon vs. dry seasons. Overall, our findings support a broad body of epidemiological evidence that community-level sanitation coverage can influence child health and environmental contamination.

We aimed to capture many potential definitions of community-level sanitation coverage, including the relevant sanitation metric and the area defined by “community”. Previous studies have measured community-level sanitation as the proportion of households within a given area with access to any latrine (Andrés et al., 2017; Harris et al., 2017; Larsen et al., 2017; Oswald et al., 2019), a basic latrine (Berendes et al., 2020), or an improved latrine (Andrés et al., 2017; Berendes et al., 2018; Fuller et al., 2016; Garn et al., 2018; Komarulzaman et al., 2017; Huda et al., 2019), with no significant differences in study results by latrine quality. In this study, we modeled both access to any type of latrine as well as access to hygienic latrines, which allowed us to assess the role of latrine quality in community-level coverage. Our definition for hygienic latrine captured both the type of latrine (improved facility) and whether it was observed to effectively isolate feces from the environment (not draining into the environment and feces well-contained in pit). For hygienic latrine coverage, we were able to assess the impact of reaching 100% community-level coverage as a binary exposure, as well as the impact of incremental increases in coverage as a continuous exposure. We were only able to assess any latrine coverage as a binary variable (100% vs. <100%) because most compounds within the specified radii had a latrine (Fig. 1). Our chosen exposure definitions of full community coverage with any latrine or with hygienic latrines capture the goals of many sanitation programs. In areas with lower or more variable latrine coverage, associations between complete coverage, fecal contamination, and child health might be stronger than what we measured here.

Although most associations we observed in our analysis were small, we found that 100% community-level coverage with any latrine was more important for fecal contamination and infectious disease transmission than 100% community-level coverage with exclusively hygienic latrines. This might indicate that access to any latrine may effectively reduce community contamination, for example by reducing open defecation. It is also possible that latrines classified as hygienic in our analysis did not sufficiently differ from unhygienic latrines in their ability to isolate fecal waste from the environment. For example, we did not collect data on pit emptying practices among neighboring compounds, and unsafe emptying or disposal practices could attenuate the benefits of community-level coverage with hygienic latrines. Among target households that reported emptying their latrine pit during the study period, 69% reported burying the pit contents, while 31% disposed of pit contents in a body of water or field. We also did not measure actual latrine use among neighbors and cannot ascertain whether the presence of a latrine or a hygienic latrine in the compound indicated exclusive use of these facilities for defecation. Among target households in our study, 77% reported exclusive latrine use among adults. Since these households were in the control arm of the parent trial and received no intervention, their practices are likely representative of neighboring compounds. Future research on community-level sanitation should aim to capture more nuanced dimensions of latrine use and pit emptying.

Definitions of “community” or “neighborhood” in previous literature have varied considerably, sometimes as entire villages or sampling clusters, which can encompass multiple villages (Berendes et al., 2017, 2020; Fuller and Eisenberg, 2016; Garn et al., 2018; Kmush et al., 2021; Komarulzaman et al., 2017; Larsen et al., 2017; Oswald et al., 2019; Vyas et al., 2016), and other times as circular areas around a target household defined by a set radius ranging between 20-1,000 m (Berendes et al., 2018; Fuller et al., 2016; Harris et al., 2017; Huda et al., 2019). We chose a maximum radius of 100 m in this study to focus on proximate fecal contamination amongst nearby neighbors and assessed a smaller radius (50 m) to assess differences in transmission by distance. Our results were qualitatively similar between 50 m and 100 m exposures, although associations were generally stronger at 50 m, suggesting that the relevant distance for pathogen transport from latrines in this setting was captured within this smaller radius. Another study similarly found that community sanitation within 50 m but not 100 m was associated with fecal contamination (Berendes et al., 2018), while a different study found no association between community-level coverage within 20 m and fecal contamination (Huda et al., 2019). Most of the research on community-level sanitation has measured coverage over large sampling areas, and almost all these studies found that coverage was associated with improved child health. Pathogens introduced into the environment through unsafe sanitation may be carried by people, animals, food, and water as they travel across and between communities over large areas. Our analysis did not capture any potential roles of community-level sanitation coverage over larger scales. However, analyses at larger scales likewise fail to capture relevant associations within more narrowly defined communities (e.g., close-range neighbors) and may also miss heterogeneity in sanitation coverage within larger geographical units. Research on community-level sanitation coverage should more clearly differentiate between broadly defined communities comprised of entire villages, neighborhoods, or more, and narrowly defined communities of proximate neighbors. It is possible that sanitation coverage is important at each of these scales in unique ways and assessing coverage at only one scale might provide an incomplete picture and miss interactions across scales. Systems-based approaches to studying enteric disease transmission may be needed to simultaneously capture the role of community-level sanitation coverage across its numerous dimensions and their interactions (Eisenberg et al., 2012).

Population density is also relevant for understanding community-level sanitation. In our rural Bangladeshi setting, population density ranged from 0 to 354 individuals within 100 m (median = 82), and the importance of community-level sanitation coverage increased with increasing population density. In our analysis, compounds in relatively high-density areas whose neighbors within 50 m all had at least one latrine had up to 0.25-log lower *E. coli* counts on hands and 33% lower prevalence of child diarrhea. This relationship may be stronger in urban settings with higher population density, although generalizability to urban settings is limited due to differences in the types of sanitation infrastructure typically used in each setting. Population density should always be considered in measures of community-level sanitation coverage.

Among environmental samples, we found statistically significant associations between community-level coverage and *E.coli* counts in stored water and on child and caregiver hand rinses, but found no associations in soil or food samples. It is possible that our smaller sample size for soil and food samples, which were only collected during two of eight sampling rounds, was not sufficient to detect associations for these sample types. It is also possible that contamination of soil and food is more strongly connected to practices within the household (e.g., child defecation, animal feces management, food hygiene) than community-level sanitation. In contrast, contamination in drinking water might be connected to community sanitation through contamination occurring at the source (primarily tubewells in this setting) by groundwater infiltration or surface runoff into the wellhead. While domestic activities are an important determinant of hand contamination (Pickering et al., 2011), community-level sanitation might also impact hand contamination through person-to-person contact with community members. Overall, small magnitudes of the associations for water and hand rinses and small sample size for soil and food in our analysis preclude a robust understanding of the important pathways between community-level sanitation and household environmental contamination.

Associations between community-level sanitation and diarrheal disease were stronger than those for *E. coli* contamination, possibly because *E. coli* presence does not correlate strongly with pathogen contamination levels (Goddard et al., 2020). Community sanitation coverage may also improve health through pathways not captured in our study, such as reducing pathogen transmission by flies. Previous studies have found that community-level latrine coverage is associated with larger reductions in child diarrhea than household-level latrine access (Andrés et al., 2017; Harris et al., 2017; Komarulzaman et al., 2017). The sanitation intervention in the parent WASH Benefits trial that provided latrine upgrades to compounds was associated with a 39% relative reduction (PR = 0.61, 95% CI 0.46, 0.81) in child diarrhea compared to controls (Luby et al., 2018). In a previous analysis of our longitudinal substudy nested within the sanitation and control arms of the parent trial, we estimated a 19% relative reduction (PR = 0.81, 95% CI 0.66, 1.00) in diarrhea prevalence compared to controls (Contreras et al., 2022). The intervention also led to a 0.08-log reduction in *E. coli* counts in stored water and on child hands compared to controls, measured 1–3.5 years after implementation, but did not reduce *E. coli* in environmental samples at earlier sampling timepoints (Contreras et al., 2021; Ercumen et al., 2018). While the present observational analysis is susceptible to unmeasured confounding, our findings indicate some associations with community-level coverage that are comparable to or larger in size than the effects of the compound-level intervention, especially in areas with high population density.

One potential limitation of our study was the approximation of a 100 m radius based on ∼300 steps from the study compound. If field staff did not travel a full 100 m, we may have missed neighbors that were within range. We used GPS data to exclude any neighboring compounds field staff visited that were beyond 100 m. In addition, our analysis was observational, and community-level sanitation coverage appeared highly associated with socioeconomic status. Although we adjusted for socioeconomic factors, including maternal education and wealth, it is possible that residual confounding was present. We analyzed several forms of the exposure variables, which increased the probability that one or more significant results were due to chance. However, the trends we observed are biologically plausible, such as more pronounced protective associations with increasing community-level coverage in high-density areas and within shorter pathogen transport distances. Also, our analysis was pre-specified (except effect modification by season) and highly powered to detect small reductions in *E. coli* counts with statistical precision due to the large number of samples collected and child observations made over eight data collection rounds. Generalizability of our findings to other settings may be limited due to the context-dependent designs of sanitation systems and hydrogeological features that can influence the environmental transport of pathogens, such as high groundwater table and a lithology dominated by alluvial sediments in our study area (Ahmed et al., 2004).

## 5 Conclusions

Overall, we found that latrine coverage within highly proximate areas was associated with reduced environmental fecal contamination and improved child health, especially where population density is relatively high in this rural setting. The associations between community-level sanitation coverage and environmental contamination and diarrhea were sensitive to how coverage was defined. There is no current consensus on the relevant scale over which community-level coverage influences fecal contamination or health, and few studies have assessed the role of population density. Continued work is needed to understand the complexities of pathogen transmission across multiple scales of community, which can inform policy for implementing transformative sanitation systems that effectively protect community health.

## Funding sources

10.13039/100000865Bill & Melinda Gates Foundation, 10.13039/100000002National Institutes of Health (NIH).

## Data statement

De-identified data used for this analysis will be made freely available on OSF upon publication (https://osf.io/6u7cn/).
