# Supplementary material for: Influence of community-level sanitation coverage and population density on environmental fecal contamination and child health in a longitudinal cohort in rural Bangladesh
Source: Int J Hyg Environ Health. 2022 Aug;245:114031. doi: 10.1016/j.ijheh.2022.114031 (PMC9489923; doi:10.1016/j.ijheh.2022.114031)
Supplement: Multimedia component 1 [file mmc1.docx]

**Supplementary Material:**

**Influence of community-level sanitation coverage and population density on environmental fecal contamination and child health in a longitudinal cohort in rural Bangladesh**

Jesse D. Contreras^1^, Mahfuza Islam^2^, Andrew Mertens^3^, Amy J. Pickering^4^, Laura H. Kwong^5^, Benjamin F. Arnold^6^, Jade Benjamin-Chung^7^, Alan E. Hubbard^3^, Mahfuja Alam^2^, Debashis Sen^2^, Sharmin Islam^2^, Mahbubur Rahman^2^, Leanne Unicomb^2^, Stephen P. Luby^8^,

John M. Colford, Jr. ^3^, and Ayse Ercumen^1*^

1: Department of Forestry and Environmental Resources, North Carolina State University; Raleigh, North Carolina, 27695, United States

2: Environmental Interventions Unit, Infectious Disease Division, International Centre for Diarrhoeal Disease Research Bangladesh; Dhaka, 1212, Bangladesh

3: Division of Epidemiology and Biostatistics, School of Public Health, University of California, Berkeley; Berkeley, California, 94720, United States

4: Department of Civil and Environmental Engineering, University of California, Berkeley; Berkeley, California, 94720, United States

5: Division of Environmental Health Sciences, School of Public Health, University of California, Berkeley; Berkeley, California, 94720, United States

6: Francis I. Proctor Foundation, University of California, San Francisco; San Francisco, California, 94158, United States

7: Department of Epidemiology and Population Health, Stanford University; Palo Alto, California, 94304, United States

8: Woods Institute for the Environment, Stanford University; Stanford, California, 94305, United States

*Corresponding Author: Ayse Ercumen, Jordan Hall Addition 2225, Raleigh, NC, 27606,

+1 (510) 225 8828, [aercume@ncsu.edu](mailto:aercume@ncsu.edu)

Funding Sources: Bill & Melinda Gates Foundation, National Institutes of Health (NIH)

Table of Contents

Table S1: Bivariate associations between potential covariates and study outcomes. Potential covariates that were associated with an outcome (alpha = 0.20) were included in all adjusted models for that outcome. 5

Table S2: Residual spatial correlation between study clusters for each outcome of interest. Moran’s I calculated on residuals of adjusted models using a continuous form hygienic latrine coverage. 9

*Primary Regression Results*

Table S3: Adjusted and unadjusted associations between study outcomes and community-level any latrine coverage (the proportion of compounds within range with at least one latrine), modeled as a binary variable (100% vs. <100%). Exposures were modeled for two different radii (50 m and 100 m) around study compounds. All models include robust standard errors. 10

Table S4: Adjusted and unadjusted associations between study outcomes and community-level hygienic latrine coverage (the proportion of compounds within range with only hygienic latrines), modeled as a binary variable (100% vs. <100%). Exposures were modeled for two different radii (50 m and 100 m) around study compounds. All models include robust standard errors. 11

*Sensitivity Analyses: Outcomes Restricted to Second Sampling Round*

Table S5: Sensitivity analysis: adjusted and unadjusted associations between study outcomes measured during the second sampling round only and community-level any latrine coverage (the proportion of compounds within range with at least one latrine), modeled as a binary variable (100% vs. <100%). All models include robust standard errors. 12

Table S6: Sensitivity analysis: adjusted and unadjusted associations between study outcomes measured during the second sampling round only and community-level hygienic latrine coverage (the proportion of compounds within range with only hygienic latrines), modeled as a binary variable (100% vs. <100%). All models include robust standard errors. 13

Table S7: Sensitivity analysis: adjusted and unadjusted associations between study outcomes measured during the second sampling round only and community-level hygienic latrine coverage (the proportion of compounds within range with only hygienic latrines), modeled as a continuous variable. Estimates reflect changes in study outcomes associated with a 10 percentage point increase in hygienic latrine coverage. All models include robust standard errors. 14

*Effect Modification by Population Density*

Table S8: Analysis of effect modification by population density on community any latrine coverage (the proportion of compounds within range with at least one latrine) within 50 m of study compounds. Exposure modeled as a binary variable (100% coverage vs. <100%). All models are adjusted and include robust standard errors. 15

Table S9: Analysis of effect modification by population density on community hygienic latrine coverage (the proportion of compounds within range with only hygienic latrines) within 50 m of study compounds. Exposure modeled as a binary variable (100% coverage vs. <100%). All models are adjusted and include robust standard errors. 16

Figure S1: Effect modification by population density on community-level any latrine coverage (the proportion of compounds within range with at least one latrine) and community hygienic latrine coverage (the proportion of compounds within range with only hygienic latrines) within 100 m of study compounds and study outcomes. Exposures were modeled as binary variables (100% vs. <100% coverage). Plots show subgroup estimates within tertiles of population density. P-values are for the interaction term between continuous population density and the exposure. All models are adjusted and include robust standard errors. 18

Table S10: Analysis of effect modification by population density on community any latrine coverage (the proportion of compounds within range with at least one latrine) within 100 m of study compounds. Exposure modeled as a binary variable (100% coverage vs. <100%). All models are adjusted and include robust standard errors. 19

Table S11: Analysis of effect modification by population density on community hygienic latrine coverage (the proportion of compounds within range with only hygienic latrines) within 100 m of study compounds. Exposure modeled as a binary variable (100% coverage vs. <100%). All models are adjusted and include robust standard errors. 21

Table S12: Analysis of effect modification by population density on community hygienic latrine coverage (the proportion of compounds within range with only hygienic latrines) within 50 m of study compounds. Exposure modeled as a continuous variable in 10 percentage point increments. All models are adjusted and include robust standard errors. 23

Table S13: Analysis of effect modification by population density on community hygienic latrine coverage (the proportion of compounds within range with only hygienic latrines) within 100 m of study compounds. Exposure modeled as a continuous variable in 10 percentage point increments. All models are adjusted and include robust standard errors. 24

*Effect Modification by Season*

Table S14: Analysis of effect modification by season (monsoon vs. dry) on community any latrine coverage (the proportion of compounds within range with at least one latrine) within 50 m of study compounds. Exposure modeled as a binary variable (100% coverage vs. <100%). All models are adjusted and include robust standard errors. 25

Table S15: Analysis of effect modification by season (monsoon vs. dry) on community hygienic latrine coverage (the proportion of compounds within range with only hygienic latrines) within 50 m of study compounds. Exposure modeled as a binary variable (100% coverage vs. <100%). All models are adjusted and include robust standard errors. 26

Figure S2: Effect modification by season (monsoon vs. dry) on community-level any latrine coverage (the proportion of compounds within range with at least one latrine) and community hygienic latrine coverage (the proportion of compounds within range with only hygienic latrines) within 100 m of study compounds and study outcomes. Exposures were modeled as binary variables (100% vs. <100% coverage). Plots show subgroup estimates within each season. P-values are for the interaction term between season and the exposure. All models are adjusted and include robust standard errors. 27

Table S16: Analysis of effect modification by season (monsoon vs. dry) on community any latrine coverage (the proportion of compounds within range with at least one latrine) within 100 m of study compounds. Exposure modeled as a binary variable (100% coverage vs. <100%). All models are adjusted and include robust standard errors. 28

Table S17: Analysis of effect modification by season (monsoon vs. dry) on community hygienic latrine coverage (the proportion of compounds within range with only hygienic latrines) within 100 m of study compounds. Exposure modeled as a binary variable (100% coverage vs. <100%). All models are adjusted and include robust standard errors. 29

Table S18: Analysis of effect modification by season (monsoon vs. dry) on community hygienic latrine coverage (the proportion of compounds within range with only hygienic latrines) within 50 m of study compounds. Exposure modeled as a continuous variable in 10 percentage point increments. All models are adjusted and include robust standard errors. 30

Table S19: Analysis of effect modification by season (monsoon vs. dry) on community hygienic latrine coverage (the proportion of compounds within range with only hygienic latrines) within 100 m of study compounds. Exposure modeled as a continuous variable in 10 percentage point increments. All models are adjusted and include robust standard errors. 31

# **Table S1:** Bivariate associations between potential covariates and study outcomes. Potential covariates that were associated with an outcome (alpha = 0.20) were included in all adjusted models for that outcome. Note: -0.00 indicates a value less than -0.01 that rounded to 0.00.

| Potential Covariate | Difference in log_10_ *E. coli* Counts (95% CI) | | | | | Prevalence Difference (95% CI) | |
| --- | --- | --- | --- | --- | --- | --- | --- |
|  | Stored Water | Child Hand Rinses | Caregiver Hand Rinses | Soil | Food | Diarrheal Disease | Acute Respiratory Infection |
| Maternal years of education | -0.04 (-0.05, -0.03)* | -0.04 (-0.06, -0.03)* | -0.04 (-0.05, -0.03)* | -0.05 (-0.08, -0.02)* | -0.02 (-0.07, 0.03) | -0.00 (-0.00, 0.00) | -0.00 (-0.00, 0.00) |
| Food insecurity | 0.09 (0.04, 0.14)* | 0.09 (0.04, 0.13)* | 0.08 (0.04, 0.12)* | 0.02 (-0.10, 0.14) | 0.09 (-0.09, 0.27) | 0.03 (0.01, 0.04)* | 0.02 (0.00, 0.03)* |
| *Food secure* | -- | -- | -- | -- | -- | -- | -- |
| *Mildly food insecure* | -- | -- | -- | -- | -- | -- | -- |
| *Moderately food insecure* | -- | -- | -- | -- | -- | -- | -- |
| *Severely food insecure* | -- | -- | -- | -- | -- | -- | -- |
| Wealth (quartiles 1-4) | -0.16 (-0.28, -0.03)* | -0.23 (-0.34, -0.12)* | -0.16 (-0.27, -0.05)* | -0.31 (-0.61, -0.00)* | -0.05 (-0.53, 0.43) | 0.02 (-0.01, 0.05)* | -0.04 (-0.08, -0.01)* |
| Month of measurement | -0.16 (-2.18, 1.85) | 1.04 (-0.93, 3.00)* | -0.47 (-2.47, 1.52) | 1.18 (0.82, 1.53)* | 1.06 (0.50, 1.62)* | 0.18 (-0.31, 0.66) | 0.25 (-0.33, 0.83) |
| Most recent time it rained | -0.12 (-0.14, -0.09* | -0.01 (-0.04, 0.01) | -0.01 (-0.04, 0.01) | 0.05 (-0.02, 0.13)* | -0.05 (-0.17, 0.06) | -0.00 (-0.01, 0.00)* | 0.01 (-0.00, 0.01)* |
| *Currently raining* | -- | -- | -- | -- | -- | -- | -- |
| *Rained earlier today* | -- | -- | -- | -- | -- | -- | -- |
| *Rained yesterday* | -- | -- | -- | -- | -- | -- | -- |
| *Rained the day before yesterday* | -- | -- | -- | -- | -- | -- | -- |
| *Rained in the last week* | -- | -- | -- | -- | -- | -- | -- |
| *Did not rain in the last week* | -- | -- | -- | -- | -- | -- | -- |
| Mother’s age in years (5 year increment) | 0.04 (-0.00, 0.08)* | 0.01 (-0.03, 0.05) | 0.09 (0.05, 0.13)* | 0.00 (-0.10, 0.11) | -0.07 (-0.23, 0.09) | 0.01 (0.00, 0.02)* | -0.01 (-0.02, 0.01) |
| Number of children <18 in the household | 0.05 (0.01, 0.08)* | 0.01 (-0.02, 0.04) | 0.06 (0.03, 0.09)* | 0.08 (-0.00, 0.17)* | 0.06 (-0.08, 0.20) | 0.02 (0.01, 0.03)* | 0.01 (-0.00, 0.02) |
| Number of individuals living in the household | -0.01 (-0.03, 0.01)* | -0.02 (-0.03, 0.00)* | 0.00 (-0.02, 0.02) | 0.05 (0.00, 0.10)* | -0.00 (-0.08, 0.07) | 0.00 (-0.00, 0.01) | -0.00 (-0.00, 0.01) |
| Distance in minutes to household’s primary drinking water source | 0.10 (0.06, 0.13)* | 0.06 (0.03, 0.09)* | 0.06 (0.03, 0.09)* | 0.11 (0.03, 0.19)* | 0.01 (-0.12, 0.13) | 0.00 (-0.00, 0.01) | 0.00 (-0.01, 0.01) |
| Improved roof | -0.01 (-0.37, 0.35) | -0.03 (-0.37, 0.31) | 0.12 (-0.21, 0.46) | -0.58 (-1.44, 0.29)* | -0.03 (-1.39, 1.32) | 0.06 (-0.03, 0.15)* | 0.06 (-0.05, 0.17) |
| Improved floor | -0.20 (-0.33, -0.06)* | -0.28 (-0.40, -0.16)* | -0.15 (-0.27, -0.03)* | -0.17 (-0.52, 0.19) | -0.10 (-0.65, 0.44) | -0.02 (-0.05, 0.01) | -0.07 (-0.11, -0.04)* |
| Improved walls | 0.04 (-0.05, 0.13) | 0.02 (-0.06, 0.10) | 0.02 (-0.06, 0.10) | 0.22 (-0.00, 0.43)* | -0.25 (-0.58, 0.07)* | 0.01 (-0.02, 0.03) | 0.02 (-0.01, 0.04) |
| Primary latrine used by index household is hygienic | -0.14 (-0.24, -0.04)* | -0.05 (-0.15, 0.04) | -0.05 (-0.15, 0.04) | -0.07 (-0.33, 0.19) | 0.10 (-0.30, 0.49) | -0.01 (-0.04, 0.01) | -0.02 (-0.04, 0.01) |
| Number of cattle | 0.12 (0.08, 0.16)* | 0.05 (0.01, 0.08)* | 0.11 (0.07, 0.14)* | 0.20 (0.10, 0.29)* | 0.01 (-0.14, 0.15) | -0.00 (-0.01, 0.01) | 0.00 (-0.01, 0.02) |
| *None* | -- | -- | -- | -- | -- | -- | -- |
| *Tertile 1 (1-2)* | -- | -- | -- | -- | -- | -- | -- |
| *Tertile 2 (3-4)* | -- | -- | -- | -- | -- | -- | -- |
| *Tertile 3 (5-57)* | -- | -- | -- | -- | -- | -- | -- |
| Number of poultry | 0.13 (0.09, 0.18)* | 0.10 (0.06, 0.14)* | 0.09 (0.05, 0.13)* | 0.26 (0.15, 0.37)* | -0.03 (-0.20, 0.14) | -0.00 (-0.01, 0.01) | 0.01 (-0.00, 0.02)* |
| *None* | -- | -- | -- | -- | -- | -- | -- |
| *Tertile 1 (1-10)* | -- | -- | -- | -- | -- | -- | -- |
| *Tertile 2 (11-21)* | -- | -- | -- | -- | -- | -- | -- |
| *Tertile 3 (22-1203)* | -- | -- | -- | -- | -- | -- | -- |
| Number of goats and sheep | 0.05 (0.01, 0.10)* | 0.05 (0.01, 0.09)* | 0.09 (0.05, 0.13)* | 0.07 (-0.04, 0.18)* | -0.01 (-0.18, 0.16) | 0.00 (-0.01, 0.01) | 0.01 (-0.00, 0.02)* |
| *None* | -- | -- | -- | -- | -- | -- | -- |
| *Tertile 1 (1-2)* | -- | -- | -- | -- | -- | -- | -- |
| *Tertile 2 (3)* | -- | -- | -- | -- | -- | -- | -- |
| *Tertile 3 (4-20)* | -- | -- | -- | -- | -- | -- | -- |
| Number of other animals | 0.08 (0.01, 0.14)* | 0.06 (-0.00, 0.11)* | 0.02 (-0.04, 0.08) | 0.05 (-0.11, 0.21) | 0.08 (-0.17, 0.33) | 0.02 (0.00, 0.03)* | 0.01 (-0.00, 0.03)* |
| *None* | -- | -- | -- | -- | -- | -- | -- |
| *Tertile 1 (1)* | -- | -- | -- | -- | -- | -- | -- |
| *Tertile 2 (2-3)* | -- | -- | -- | -- | -- | -- | -- |
| *Tertile 3 (4-12)* | -- | -- | -- | -- | -- | -- | -- |
| Open defecation by children <3 | -0.00 (-0.00, 0.00)* | 0.00 (-0.00, 0.00) | -0.00 (-0.00, 0.00) | -0.01 (-0.03, 0.02) | -0.02 (-0.05, 0.01) | -0.00 (-0.00, 0.00) | -0.00 (-0.00, -0.00)* |
| *Daily* | -- | -- | -- | -- | -- | -- | -- |
| *Occasionally* | -- | -- | -- | -- | -- | -- | -- |
| *Never* | -- | -- | -- | -- | -- | -- | -- |
| Open defecation by children 3-8 | -0.00 (-0.00, -0.00)* | -0.00 (-0.00, -0.00)* | -0.00 (-0.00, -0.00)* | -0.00 (-0.00, 0.00) | -0.00 (-0.01, 0.00) | -0.00 (-0.00, 0.00) | -0.00 (-0.00, 0.00) |
| *Daily* | -- | -- | -- | -- | -- | -- | -- |
| *Occasionally* | -- | -- | -- | -- | -- | -- | -- |
| *Never* | -- | -- | -- | -- | -- | -- | -- |
| Type of storage container | -0.02 (-0.07, 0.03) | -- | -- | -- | -- | -- | -- |
| *Kolshi* | -- | -- | -- | -- | -- | -- | -- |
| *Jug* | -- | -- | -- | -- | -- | -- | -- |
| *Narrow mouthed container* | -- | -- | -- | -- | -- | -- | -- |
| *Wide mouthed container* | -- | -- | -- | -- | -- | -- | -- |
| Cover status of storage container | 0.12 (0.07, 0.17)* | -- | -- | -- | -- | -- | -- |
| *Fully covered* | -- | -- | -- | -- | -- | -- | -- |
| *Partially covered* | -- | -- | -- | -- | -- | -- | -- |
| *Uncovered* | -- | -- | -- | -- | -- | -- | -- |
| Treatment, filtration, or boiling of stored water | -0.57 (-1.22, 0.09)* | -- | -- | -- | -- | -- | -- |
| Source of stored drinking water | -0.10 (-0.21, 0.02)* | -- | -- | -- | -- | -- | -- |
| *Tubewell* | -- | -- | -- | -- | -- | -- | -- |
| *Dugwell* | -- | -- | -- | -- | -- | -- | -- |
| *Piped water* | -- | -- | -- | -- | -- | -- | -- |
| Length of time stored | -0.00 (-0.00, 0.00) | -- | -- | -- | -- | -- | -- |
| Observed to wash hands before sampling | -- | 0.03 (-0.03, 0.09) | -0.13 (-0.17, -0.10)* | -- |  |  |  |
| Time of collection (minutes after 7am) | -- | -0.00 (-0.00, 0.00)* | -0.00 (-0.00, -0.00)* | -0.00 (-0.00, 0.00) | -- | -- | -- |
| Age of child (months) | -- | -0.00 (-0.01, 0.00) | -- | -- | -- | -0.00 (-0.00, -0.00)* | -0.00 (-0.00, -0.00)* |
| Sex of child (female vs. male) | -- | -0.13 (-0.20, -0.05)* | -- | -- | -- | -0.04 (-0.06, -0.02)* | -0.03 (-0.05, -0.00)* |
| Sample collected in sunlight vs. shaded area | -- | -- | -- | 0.01 (-0.01, 0.03) | -- | -- | -- |
| Visible wetness of sampling area | -- | -- | -- | 0.06 (-0.16, 0.28) | -- | -- | -- |
| Measured moisture content | -- | -- | -- | 0.05 (0.04, 0.07)* | -- | -- | -- |
| Type of food item | -- | -- | -- | -- | 0.10 (-0.27, 0.48) | -- | -- |
| *Rice* | -- | -- | -- | -- | -- | -- | -- |
| *Khichuri* | -- | -- | -- | -- | -- | -- | -- |
| *Gruel/porridge* | -- | -- | -- | -- | -- | -- | -- |
| Length of time stored (hours) | -- | -- | -- | -- | 0.11 (0.08, 0.14)* | -- | -- |
| Food transferred | -- | -- | -- | -- | 0.03 (-0.00, 0.06)* | -- | -- |
| *With utensil* | -- | -- | -- | -- | -- | -- | -- |
| *With hands* | -- | -- | -- | -- | -- | -- | -- |
| *Poured directly from pot* | -- | -- | -- | -- | -- | -- | -- |
| Cover status of food container | -- | -- | -- | -- | 0.24 (0.00, 0.48)* | -- | -- |
| *Fully covered* | -- | -- | -- | -- | -- | -- | -- |
| *Partially covered* | -- | -- | -- | -- | -- | -- | -- |
| *Uncovered* | -- | -- | -- | -- | -- | -- | -- |
| Where food was stored | -- | -- | -- | -- | -0.05 (-0.25, 0.16) | -- | -- |
| *On the ground* | -- | -- | -- | -- | -- | -- | -- |
| *On a table* | -- | -- | -- | -- | -- | -- | -- |
| *In a meatsafe/cabinet* | -- | -- | -- | -- | -- | -- | -- |
| *In a shika* | -- | -- | -- | -- | -- | -- | -- |
| Temperature in food storage area | -- | -- | -- | -- | 0.02 (-0.06, 0.10) | -- | -- |
| Humidity in food storage area | -- | -- | -- | -- | 0.02 (0.00, 0.04)* | -- | -- |
| Temperature of food in storage container | -- | -- | -- | -- | -0.00 (-0.01, 0.01) | -- | -- |
| *p-value < 0.20 | | | | | | | |

# **Table S2:** Residual spatial correlation between study clusters for each outcome of interest. Moran’s I calculated on residuals of adjusted models using a continuous form hygienic latrine coverage.

| **Outcome** | **p-Value of Moran’s I** |
| --- | --- |
| *Log10 E. coli MPN Differences* | -- |
| Stored Water | .82 |
| Child Hand Rinses | .46 |
| Caregiver Hand Rinses | .28 |
| Soil | .97 |
| Food | .35 |
| *Prevalence Ratios* | -- |
| Diarrheal Disease | .47 |
| Acute Respiratory Infection (ARI) | .20 |

# **Table S3:** Adjusted and unadjusted associations between study outcomes and community-level any latrine coverage (the proportion of compounds within range with at least one latrine), modeled as a binary variable (100% vs. <100%). Exposures were modeled for two different radii (50 m and 100 m) around study compounds. All models include robust standard errors.

|  | 50 m | | | | 100 m | | | |
| --- | --- | --- | --- | --- | --- | --- | --- | --- |
|  | Unadjusted | | Adjusted^ψ^ | | Unadjusted | | Adjusted^ψ^ | |
|  | *n* | Estimate | *n* | Estimate | *n* | Estimate | *n* | Estimate |
| ***Log10 E. coli MPN Differences*** | -- | -- | -- | -- | -- | -- | -- | -- |
| Stored Water | 2210 | -0.20 (-0.32, -0.07)* | 2129 | -0.13 (-0.26, -0.01)* | 2306 | -0.11 (-0.23, 0.00) | 2221 | -0.04 (-0.14, 0.05) |
| Child Hand Rinses | 2495 | -0.18 (-0.31, -0.04)* | 2450 | -0.13 (-0.24, -0.02)* | 2605 | -0.12 (-0.22, -0.02)* | 2556 | -0.06 (-0.14, 0.03) |
| Caregiver Hand Rinses | 2529 | -0.21 (-0.36, -0.05)* | 2493 | -0.16 (-0.29, -0.03)* | 2640 | -0.15 (-0.27, -0.04)* | 2601 | -0.10 (-0.21, 0.00) |
| Soil | 364 | 0.00 (-0.23, 0.22) | 359 | 0.17 (-0.02, 0.37) | 383 | -0.06 (-0.30, 0.19) | 378 | 0.17 (-0.04, 0.38) |
| Food | 265 | 0.18 (-0.14, 0.50) | 265 | 0.14 (-0.18, 0.47) | 273 | 0.11 (-0.22, 0.44) | 273 | 0.14 (-0.16, 0.45) |
| ***Prevalence Ratios*** | -- | -- | -- | -- | -- | -- | -- | -- |
| Diarrheal Disease | 4461 | 0.81 (0.64, 1.04) | 4426 | 0.82 (0.64, 1.04) | 4662 | 0.83 (0.67, 1.04) | 4627 | 0.83 (0.67, 1.02) |
| Acute Respiratory Infection (ARI) | 4464 | 0.82 (0.68, 0.98)* | 4429 | 0.84 (0.69, 1.03) | 4665 | 0.91 (0.77, 1.08) | 4630 | 0.92 (0.78, 1.09) |
| ***Prevalence Differences*** | -- | -- | -- | -- | -- | -- | -- | -- |
| Diarrheal Disease | 4461 | -0.03 (-0.07, 0.01) | 4426 | -0.03 (-0.07, 0.01) | 4662 | -0.03 (-0.06, 0.01) | 4627 | -0.03 (-0.06, 0.01) |
| Acute Respiratory Infection (ARI) | 4464 | -0.05 (-0.10, 0.00)* | 4429 | -0.04 (-0.09, 0.01) | 4665 | -0.02 (-0.06, 0.02) | 4630 | -0.02 (-0.06, 0.02) |
| ^ψ^*See Table S1 for full list of potential and selected covariates by outcome*  **Statistically significant at the 95% level* | | | | | | | | |

# **Table S4:** Adjusted and unadjusted associations between study outcomes and community-level hygienic latrine coverage (the proportion of compounds within range with only hygienic latrines), modeled as a binary variable (100% vs. <100%). Exposures were modeled for two different radii (50 m and 100 m) around study compounds. All models include robust standard errors.

|  | 50 m | | | | 100 m | | | |
| --- | --- | --- | --- | --- | --- | --- | --- | --- |
|  | Unadjusted | | Adjusted^ψ^ | | Unadjusted | | Adjusted^ψ^ | |
|  | *n* | Estimate | *n* | Estimate | *n* | Estimate | *n* | Estimate |
| ***Log10 E. coli MPN Differences*** | -- | -- | -- | -- | -- | -- | -- | -- |
| Stored Water | 2204 | -0.02 (-0.16, 0.13) | 2123 | 0.03 (-0.10, 0.16) | 2299 | -0.16 (-0.38, 0.05) | 2214 | -0.05 (-0.25, 0.14) |
| Child Hand Rinses | 2488 | -0.10 (-0.23, 0.03) | 2443 | -0.05 (-0.16, 0.06) | 2598 | -0.08 (-0.26, 0.09) | 2550 | -0.01 (-0.17, 0.15) |
| Caregiver Hand Rinses | 2522 | 0.02 (-0.12, 0.16) | 2486 | 0.07 (-0.06, 0.19) | 2633 | -0.06 (-0.28, 0.16) | 2594 | 0.03 (-0.15, 0.20) |
| Soil | 364 | -0.26 (-0.57, 0.06) | 359 | 0.14 (-0.12, 0.41) | 382 | -0.26 (-0.67, 0.14) | 377 | 0.23 (-0.16, 0.62) |
| Food | 265 | 0.17 (-0.23, 0.57) | 265 | 0.18 (-0.16, 0.53) | 273 | 0.08 (-0.45, 0.62) | 273 | 0.02 (-0.41, 0.46) |
| ***Prevalence Ratios*** | -- | -- | -- | -- | -- | -- | -- | -- |
| Diarrheal Disease | 4454 | 1.14 (0.85, 1.53) | 4419 | 1.13 (0.88, 1.45) | 4652 | 1.12 (0.70, 1.79) | 4617 | 1.09 (0.78, 1.52) |
| Acute Respiratory Infection (ARI) | 4457 | 0.87 (0.72, 1.05) | 4422 | 0.92 (0.77, 1.11) | 4655 | 0.77 (0.56, 1.05) | 4620 | 0.80 (0.58, 1.09) |
| ***Prevalence Differences*** | -- | -- | -- | -- | -- | -- | -- | -- |
| Diarrheal Disease | 4454 | 0.02 (-0.03, 0.07) | 4419 | 0.02 (-0.02, 0.06) | 4652 | 0.02 (-0.06, 0.09) | 4617 | 0.02 (-0.04, 0.08) |
| Acute Respiratory Infection (ARI) | 4457 | -0.03 (-0.07, 0.01) | 4422 | -0.02 (-0.06, 0.02) | 4655 | -0.05 (-0.11, 0.00) | 4620 | -0.05 (-0.11, 0.01) |
| ^ψ^*See Table S1 for full list of potential and selected covariates by outcome*  **Statistically significant at the 95% level* | | | | | | | | |

# **Table S5:** Sensitivity analysis: adjusted and unadjusted associations between study outcomes measured during the second sampling round only and community-level any latrine coverage (the proportion of compounds within range with at least one latrine), modeled as a binary variable (100% vs. <100%). All models include robust standard errors.

|  | 100 m | | | |
| --- | --- | --- | --- | --- |
|  | Unadjusted | | Adjusted^ψ^ | |
|  | *n* | Estimate | *n* | Estimate |
| ***Log10 E. coli MPN Differences*** | -- | -- | -- | -- |
| Stored Water | 295 | -0.06 (-0.25, 0.13) | 288 | -0.03 (-0.23, 0.17) |
| Child Hand Rinses | 351 | -0.08 (-0.29, 0.12) | 349 | -0.06 (-0.25, 0.14) |
| Caregiver Hand Rinses | 351 | -0.25 (-0.46, -0.03)* | 351 | -0.21 (-0.41, -0.01)* |
| ***Prevalence Ratios*** | -- | -- | -- | -- |
| Diarrheal Disease | 633 | 0.73 (0.49, 1.08) | 631 | 0.73 (0.50, 1.07) |
| Acute Respiratory Infection (ARI) | 636 | 0.96 (0.73, 1.27) | 634 | 0.97 (0.74, 1.27) |
| ***Prevalence Differences*** | -- | -- | -- | -- |
| Diarrheal Disease | 633 | -0.05 (-0.11, 0.01) | 631 | -0.05 (-0.11, 0.01) |
| Acute Respiratory Infection (ARI) | 636 | -0.01 (-0.10, 0.08) | 634 | -0.01 (-0.10, 0.08) |
| ^ψ^*See Table S1 for full list of potential and selected covariates by outcome*  **Statistically significant at the 95% level* | | | | |

# **Table S6:** Sensitivity analysis: adjusted and unadjusted associations between study outcomes measured during the second sampling round only and community-level hygienic latrine coverage (the proportion of compounds within range with only hygienic latrines), modeled as a binary variable (100% vs. <100%). All models include robust standard errors.

|  | 100 m | | | |
| --- | --- | --- | --- | --- |
|  | Unadjusted | | Adjusted^ψ^ | |
|  | *n* | Estimate | *n* | Estimate |
| ***Log10 E. coli MPN Differences*** | -- | -- | -- | -- |
| Stored Water | 294 | -0.16 (-0.54, 0.22) | 287 | -0.02 (-0.42, 0.38) |
| Child Hand Rinses | 350 | -0.06 (-0.45, 0.34) | 348 | -0.08 (-0.49, 0.33) |
| Caregiver Hand Rinses | 350 | -0.09 (-0.46, 0.28) | 350 | -0.03 (-0.38, 0.31) |
| ***Prevalence Ratios*** | -- | -- | -- | -- |
| Diarrheal Disease | 631 | 0.72 (0.28, 1.81) | 629 | 0.71 (0.31, 1.62) |
| Acute Respiratory Infection (ARI) | 634 | 0.71 (0.36, 1.40) | 632 | 0.72 (0.36, 1.44) |
| ***Prevalence Differences*** | -- | -- | -- | -- |
| Diarrheal Disease | 631 | -0.05 (-0.16, 0.06) | 629 | -0.05 (-0.15, 0.06) |
| Acute Respiratory Infection (ARI) | 634 | -0.09 (-0.25, 0.06) | 632 | -0.10 (-0.26, 0.07) |
| ^ψ^*See Table S1 for full list of potential and selected covariates by outcome*  **Statistically significant at the 95% level* | | | | |

# **Table S7:** Sensitivity analysis: adjusted and unadjusted associations between study outcomes measured during the second sampling round only and community-level hygienic latrine coverage (the proportion of compounds within range with only hygienic latrines), modeled as a continuous variable. Estimates reflect changes in study outcomes associated with a 10 percentage point increase in hygienic latrine coverage. All models include robust standard errors.

|  | 100 m | | | |
| --- | --- | --- | --- | --- |
|  | Unadjusted | | Adjusted^ψ^ | |
|  | *n* | Estimate | *n* | Estimate |
| ***Log10 E. coli MPN Differences*** | -- | -- | -- | -- |
| Stored Water | 294 | -0.03 (-0.08, 0.01) | 287 | -0.02 (-0.06, 0.03) |
| Child Hand Rinses | 350 | -0.03 (-0.07, 0.02) | 348 | -0.03 (-0.08, 0.03) |
| Caregiver Hand Rinses | 350 | -0.04 (-0.08, 0.01) | 350 | -0.03 (-0.07, 0.02) |
| ***Prevalence Ratios*** | -- | -- | -- | -- |
| Diarrheal Disease | 631 | 0.97 (0.89, 1.05) | 629 | 0.97 (0.90, 1.06) |
| Acute Respiratory Infection (ARI) | 634 | 1.00 (0.94, 1.05) | 632 | 1.01 (0.95, 1.07) |
| ***Prevalence Differences*** | -- | -- | -- | -- |
| Diarrheal Disease | 631 | -0.01 (-0.02, 0.01) | 629 | 0.00 (-0.02, 0.01) |
| Acute Respiratory Infection (ARI) | 634 | 0.00 (-0.02, 0.02) | 632 | 0.00 (-0.02, 0.02) |
| ^ψ^*See Table S1 for full list of potential and selected covariates by outcome*  **Statistically significant at the 95% level* | | | | |

# **Table S8:** Analysis of effect modification by population density on community any latrine coverage (the proportion of compounds within range with at least one latrine) within 50 m of study compounds. Exposure modeled as a binary variable (100% coverage vs. <100%). All models are adjusted and include robust standard errors.

|  | <100% of Compounds within 50 m Have At Least One Latrine | 100% of Compounds within 50 m Have At Least One Latrine | Adjusted association between exposure and outcome within subgroup of population density | | Interaction Term p-Value (continuous form of population density) |
| --- | --- | --- | --- | --- | --- |
|  | Mean (n) or Proportion (n) of Outcome | | *n* | Estimate (95% CI) |  |
| ***Log10 E. coli MPN Differences*** | -- | -- | -- | -- | -- |
| Stored Water (mean) | -- | -- | 2129 | -- | .46 |
| *Pop. Dens. Tertile 1* | 1.05 (62) | 0.92 (520) | 566 | -0.18 (-0.77, 0.41) | -- |
| *Pop. Dens. Tertile 2* | 1.23 (162) | 1.03 (726) | 869 | -0.17 (-0.34, 0.01) | -- |
| *Pop. Dens. Tertile 3* | 1.17 (308) | 0.96 (432) | 694 | -0.09 (-0.29, 0.11) | -- |
| Child Hand Rinses (mean) | -- | -- | 2450 | -- | .86 |
| *Pop. Dens. Tertile 1* | 1.38 (67) | 1.47 (594) | 641 | 0.09 (-0.11, 0.29) | -- |
| *Pop. Dens. Tertile 2* | 1.76 (170) | 1.45 (838) | 998 | -0.31 (-0.52, -0.10)* | -- |
| *Pop. Dens. Tertile 3* | 1.62 (345) | 1.45 (481) | 811 | -0.14 (-0.32, 0.05) | -- |
| Caregiver Hand Rinses (mean) | -- | -- | 2493 | -- | .06** |
| *Pop. Dens. Tertile 1* | 1.19 (69) | 1.47 (603) | 661 | 0.19 (-0.06, 0.43) | -- |
| *Pop. Dens. Tertile 2* | 1.66 (176) | 1.42 (849) | 1015 | -0.26 (-0.48, -0.04)* | -- |
| *Pop. Dens. Tertile 3* | 1.71 (347) | 1.38 (485) | 817 | -0.25 (-0.42, -0.07)* | -- |
| ***Prevalence Ratios*** | -- | -- | -- | -- | -- |
| Diarrheal Disease (proportion cases) | -- | -- | 4426 | -- | .15** |
| *Pop. Dens. Tertile 1* | 0.10 (105) | 0.17 (958) | 1047 | 1.84 (0.94, 3.63) | -- |
| *Pop. Dens. Tertile 2* | 0.13 (324) | 0.12 (1584) | 1894 | 0.93 (0.61, 1.43) | -- |
| *Pop. Dens. Tertile 3* | 0.20 (674) | 0.13 (826) | 1485 | 0.67 (0.47, 0.95)* | -- |
| Acute Respiratory Infection (ARI) (proportion cases) | -- | -- | 4429 | -- | .96 |
| *Pop. Dens. Tertile 1* | 0.21 (105) | 0.21 (958) | 1047 | 1.09 (0.77, 1.54) | -- |
| *Pop. Dens. Tertile 2* | 0.22 (324) | 0.21 (1584) | 1898 | 0.99 (0.68, 1.44) | -- |
| *Pop. Dens. Tertile 3* | 0.30 (674) | 0.24 (826) | 1484 | 0.81 (0.61, 1.10) | -- |
| ***Prevalence Differences*** | -- | -- | -- | -- | -- |
| Diarrheal Disease (proportion cases) | -- | -- | 4426 | -- | .10** |
| *Pop. Dens. Tertile 1* | 0.10 (105) | 0.17 (958) | 1047 | 0.07 (0.01, 0.14)* | -- |
| *Pop. Dens. Tertile 2* | 0.13 (324) | 0.12 (1584) | 1894 | -0.01 (-0.07, 0.05) | -- |
| *Pop. Dens. Tertile 3* | 0.20 (674) | 0.13 (826) | 1485 | -0.06 (-0.12, -0.01)* | -- |
| Acute Respiratory Infection (ARI) (proportion cases) | -- | -- | 4429 | -- | .90 |
| *Pop. Dens. Tertile 1* | 0.21 (105) | 0.21 (958) | 1047 | 0.02 (-0.05, 0.09) | -- |
| *Pop. Dens. Tertile 2* | 0.22 (324) | 0.21 (1584) | 1898 | 0.00 (-0.08, 0.08) | -- |
| *Pop. Dens. Tertile 3* | 0.30 (674) | 0.24 (826) | 1484 | -0.05 (-0.14, 0.03) | -- |
| **Subgroup p-value <.05; ** Interaction p-value <.20* | | | | | |

# **Table S9:** Analysis of effect modification by population density on community hygienic latrine coverage (the proportion of compounds within range with only hygienic latrines) within 50 m of study compounds. Exposure modeled as a binary variable (100% coverage vs. <100%). All models are adjusted and include robust standard errors.

|  | <100% of Compounds within 50 m Have Only Hygienic Latrines | 100% of Compounds within 50 m Have Only Hygienic Latrines | Adjusted association between exposure and outcome within subgroup of population density | | Interaction Term p-Value (continuous form of population density) |
| --- | --- | --- | --- | --- | --- |
|  | Mean (n) or Proportion (n) of Outcome | | *n* | Estimate (95% CI) |  |
| ***Log10 E. coli MPN Differences*** | -- | -- | -- | -- | -- |
| Stored Water (mean) | -- | -- | 2123 | -- | .10** |
| *Pop. Dens. Tertile 1* | 0.85 (294) | 1.03 (282) | 560 | 0.18 (-0.02, 0.39) | -- |
| *Pop. Dens. Tertile 2* | 1.08 (731) | 1.05 (157) | 869 | 0.01 (-0.22, 0.24) | -- |
| *Pop. Dens. Tertile 3* | 1.06 (709) | 0.73 (31) | 694 | -0.41 (-0.90, 0.07) | -- |
| Child Hand Rinses (mean) | -- | -- | 2443 | -- | .11** |
| *Pop. Dens. Tertile 1* | 1.46 (330) | 1.49 (324) | 634 | 0.08 (-0.10, 0.27) | -- |
| *Pop. Dens. Tertile 2* | 1.54 (831) | 1.34 (177) | 998 | -0.14 (-0.33, 0.06) | -- |
| *Pop. Dens. Tertile 3* | 1.54 (791) | 1.22 (35) | 811 | -0.30 (-0.70, 0.09) | -- |
| Caregiver Hand Rinses (mean) | -- | -- | -- | -- | <.01** |
| *Pop. Dens. Tertile 1* | 1.30 (332) | 1.59 (333) | 654 | 0.26 (0.10, 0.42) | -- |
| *Pop. Dens. Tertile 2* | 1.49 (845) | 1.36 (180) | 1015 | -0.05 (-0.25, 0.15) | -- |
| *Pop. Dens. Tertile 3* | 1.53 (796) | 1.22 (36) | 817 | -0.18 (-0.53, 0.16) | -- |
| ***Prevalence Ratios*** | -- | -- | -- | -- | -- |
| Diarrheal Disease (proportion cases) | -- | -- | 4419 | -- | .01** |
| *Pop. Dens. Tertile 1* | 0.13 (512) | 0.19 (544) | 1040 | 1.38 (0.95, 2.01) | -- |
| *Pop. Dens. Tertile 2* | 0.12 (1567) | 0.13 (341) | 1894 | 1.09 (0.77, 1.55) | -- |
| *Pop. Dens. Tertile 3* | 0.16 (1442) | 0.07 (58) | 1485 | 0.44 (0.13, 1.45) | -- |
| Acute Respiratory Infection (ARI) (proportion cases) | -- | -- | 4422 | -- | .82 |
| *Pop. Dens. Tertile 1* | 0.20 (512) | 0.21 (544) | 1040 | 1.08 (0.80, 1.45) | -- |
| *Pop. Dens. Tertile 2* | 0.22 (1567) | 0.20 (341) | 1898 | 0.90 (0.66, 1.21) | -- |
| *Pop. Dens. Tertile 3* | 0.27 (1442) | 0.22 (58) | 1484 | 0.98 (0.60, 1.59) | -- |
| ***Prevalence Differences*** | -- | -- | -- | -- | -- |
| Diarrheal Disease (proportion cases) | -- | -- | 4419 | -- | <.01** |
| *Pop. Dens. Tertile 1* | 0.13 (512) | 0.19 (544) | 1040 | 0.05 (-0.01, 0.11) | -- |
| *Pop. Dens. Tertile 2* | 0.12 (1567) | 0.13 (341) | 1894 | 0.01 (-0.03, 0.06) | -- |
| *Pop. Dens. Tertile 3* | 0.16 (1442) | 0.07 (58) | 1485 | -0.09 (-0.18, -0.01)* | -- |
| Acute Respiratory Infection (ARI) (proportion cases) | -- | -- | 4422 | -- | .68 |
| *Pop. Dens. Tertile 1* | 0.20 (512) | 0.21 (544) | 1040 | 0.02 (-0.05, 0.08) | -- |
| *Pop. Dens. Tertile 2* | 0.22 (1567) | 0.20 (341) | 1898 | -0.02 (-0.08, 0.04) | -- |
| *Pop. Dens. Tertile 3* | 0.27 (1442) | 0.22 (58) | 1484 | -0.01 (-0.12, 0.11) | -- |
| **Subgroup p-value <.05; ** Interaction p-value <.20* | | | | | |

# **Figure S1:** Effect modification by population density on community-level any latrine coverage (the proportion of compounds within range with at least one latrine) and community hygienic latrine coverage (the proportion of compounds within range with only hygienic latrines) within 100 m of study compounds and study outcomes. Exposures were modeled as binary variables (100% vs. <100% coverage). Plots show subgroup estimates within tertiles of population density. P-values are for the interaction term between continuous population density and the exposure. All models are adjusted and include robust standard errors.

# **Table S10:** Analysis of effect modification by population density on community any latrine coverage (the proportion of compounds within range with at least one latrine) within 100 m of study compounds. Exposure modeled as a binary variable (100% coverage vs. <100%). All models are adjusted and include robust standard errors.

|  | <100% of Compounds within 100 m Have At Least One Latrine | 100% of Compounds within 100 m Have At Least One Latrine | Adjusted association between exposure and outcome within subgroup of population density | | Interaction Term p-Value (continuous form of population density) |
| --- | --- | --- | --- | --- | --- |
|  | Mean (n) or Proportion (n) of Outcome | | *n* | Estimate (95% CI) |  |
| ***Log10 E. coli MPN Differences*** | -- | -- | -- | -- | -- |
| Stored Water (mean) | -- | -- | 2221 | -- | .12** |
| *Pop. Dens. Tertile 1* | 1.06 (183) | 0.91 (569) | 732 | -0.13 (-0.39, 0.12) | -- |
| *Pop. Dens. Tertile 2* | 1.10 (324) | 1.04 (469) | 775 | -0.03 (-0.20, 0.15) | -- |
| *Pop. Dens. Tertile 3* | 1.10 (466) | 1.01 (295) | 714 | -0.01 (-0.17, 0.14) | -- |
| Child Hand Rinses (mean) | -- | -- | 2556 | -- | .43 |
| *Pop. Dens. Tertile 1* | 1.45 (197) | 1.47 (655) | 835 | 0.10 (-0.04, 0.24) | -- |
| *Pop. Dens. Tertile 2* | 1.64 (350) | 1.44 (522) | 857 | -0.20 (-0.37, -0.03) | -- |
| *Pop. Dens. Tertile 3* | 1.57 (544) | 1.43 (337) | 864 | -0.08 (-0.22, 0.06) | -- |
| Caregiver Hand Rinses (mean) | -- | -- | 2601 | -- | .01** |
| *Pop. Dens. Tertile 1* | 1.38 (205) | 1.49 (661) | 855 | 0.10 (-0.10, 0.30) | -- |
| *Pop. Dens. Tertile 2* | 1.61 (355) | 1.39 (527) | 871 | -0.25 (-0.42, -0.07) | -- |
| *Pop. Dens. Tertile 3* | 1.61 (549) | 1.32 (343) | 875 | -0.19 (-0.36, -0.02) | -- |
| ***Prevalence Ratios*** | -- | -- | -- | -- | -- |
| Diarrheal Disease (proportion cases) | -- | -- | 4627 | -- | <.01** |
| *Pop. Dens. Tertile 1* | 0.12 (313) | 0.13 (1088) | 1392 | 0.94 (0.62, 1.42) | -- |
| *Pop. Dens. Tertile 2* | 0.15 (654) | 0.14 (971) | 1608 | 0.96 (0.66, 1.39) | -- |
| *Pop. Dens. Tertile 3* | 0.18 (1054) | 0.14 (592) | 1627 | 0.72 (0.55, 0.95)* | -- |
| Acute Respiratory Infection (ARI) (proportion cases) | -- | -- | 4630 | -- | .83 |
| *Pop. Dens. Tertile 1* | 0.17 (313) | 0.20 (1088) | 1392 | 1.14 (0.83, 1.58) | -- |
| *Pop. Dens. Tertile 2* | 0.25 (654) | 0.24 (971) | 1609 | 0.91 (0.68, 1.21) | -- |
| *Pop. Dens. Tertile 3* | 0.25 (1054) | 0.21 (592) | 1629 | 0.89 (0.70, 1.14) | -- |
| ***Prevalence Differences*** | -- | -- | -- | -- | -- |
| Diarrheal Disease (proportion cases) | -- | -- | 4627 | -- | <.01** |
| *Pop. Dens. Tertile 1* | 0.12 (313) | 0.13 (1088) | 1392 | -0.01 (-0.06, 0.04) | -- |
| *Pop. Dens. Tertile 2* | 0.15 (654) | 0.14 (971) | 1608 | -0.01 (-0.06, 0.05) | -- |
| *Pop. Dens. Tertile 3* | 0.18 (1054) | 0.14 (592) | 1627 | -0.05 (-0.09, 0.00) | -- |
| Acute Respiratory Infection (ARI) (proportion cases) | -- | -- | 4630 | -- | .75 |
| *Pop. Dens. Tertile 1* | 0.17 (313) | 0.20 (1088) | 1392 | 0.03 (-0.03, 0.09) | -- |
| *Pop. Dens. Tertile 2* | 0.25 (654) | 0.24 (971) | 1609 | -0.03 (-0.10, 0.05) | -- |
| *Pop. Dens. Tertile 3* | 0.25 (1054) | 0.21 (592) | 1629 | -0.03 (-0.08, 0.03) | -- |
| **Subgroup p-value <.05; ** Interaction p-value <.20* | | | | | |

# **Table S11:** Analysis of effect modification by population density on community hygienic latrine coverage (the proportion of compounds within range with only hygienic latrines) within 100 m of study compounds. Exposure modeled as a binary variable (100% coverage vs. <100%). All models are adjusted and include robust standard errors.

|  | <100% of Compounds within 100 m Have Only Hygienic Latrines | 100% of Compounds within 100 m Have Only Hygienic Latrines | Adjusted association between exposure and outcome within subgroup of population density | | Interaction Term p-Value (continuous form of population density) |
| --- | --- | --- | --- | --- | --- |
|  | Mean (n) or Proportion (n) of Outcome | | *n* | Estimate (95% CI) |  |
| ***Log10 E. coli MPN Differences*** | -- | -- | -- | -- | -- |
| Stored Water (mean) | -- | -- | 2214 | -- | .49 |
| *Pop. Dens. Tertile 1* | 0.96 (601) | 0.95 (144) | 725 | 0.06 (-0.24, 0.36) | -- |
| *Pop. Dens. Tertile 2* | 1.09 (724) | 0.81 (69) | 775 | -0.13 (-0.44, 0.18) | -- |
| *Pop. Dens. Tertile 3* | 1.07 (748) | 0.55 (13) | 714 | -0.51 (-1.17, 0.15) | -- |
| Child Hand Rinses (mean) | -- | -- | 2550 | -- | .82 |
| *Pop. Dens. Tertile 1* | 1.48 (685) | 1.43 (160) | 829 | -0.01 (-0.22, 0.19) | -- |
| *Pop. Dens. Tertile 2* | 1.53 (795) | 1.42 (77) | 857 | -0.13 (-0.45, 0.18) | -- |
| *Pop. Dens. Tertile 3* | 1.52 (868) | 1.45 (13) | 864 | -0.02 (-0.61, 0.57) | -- |
| Caregiver Hand Rinses (mean) | -- | -- | -- | -- | .21 |
| *Pop. Dens. Tertile 1* | 1.46 (696) | 1.49 (163) | 848 | 0.05 (-0.13, 0.22) | -- |
| *Pop. Dens. Tertile 2* | 1.50 (805) | 1.32 (77) | 871 | -0.04 (-0.38, 0.30) | -- |
| *Pop. Dens. Tertile 3* | 1.50 (878) | 1.31 (14) | 875 | -0.15 (-0.69, 0.38) | -- |
| ***Prevalence Ratios*** | -- | -- | -- | -- | -- |
| Diarrheal Disease (proportion cases) | -- | -- | 4617 | -- | .05** |
| *Pop. Dens. Tertile 1* | 0.12 (1157) | 0.19 (234) | 1382 | 1.24 (0.78, 1.99) | -- |
| *Pop. Dens. Tertile 2* | 0.15 (1488) | 0.12 (137) | 1608 | 0.89 (0.55, 1.43) | -- |
| *Pop. Dens. Tertile 3* | 0.16 (1629) | 0.12 (17) | 1627 | 0.77 (0.24, 2.42) | -- |
| Acute Respiratory Infection (ARI) (proportion cases) | -- | -- | 4620 | -- | .49 |
| *Pop. Dens. Tertile 1* | 0.20 (1157) | 0.18 (234) | 1382 | 0.80 (0.56, 1.13) | -- |
| *Pop. Dens. Tertile 2* | 0.25 (1488) | 0.15 (137) | 1609 | 0.55 (0.35, 0.85)* | -- |
| *Pop. Dens. Tertile 3* | 0.24 (1629) | 0.29 (17) | 1629 | 1.93 (0.50, 7.43) | -- |
| ***Prevalence Differences*** | -- | -- | -- | -- | -- |
| Diarrheal Disease (proportion cases) | -- | -- | 4617 | -- | .04** |
| *Pop. Dens. Tertile 1* | 0.12 (1157) | 0.19 (234) | 1382 | 0.05 (-0.03, 0.12) | -- |
| *Pop. Dens. Tertile 2* | 0.15 (1488) | 0.12 (137) | 1608 | -0.02 (-0.07, 0.04) | -- |
| *Pop. Dens. Tertile 3* | 0.16 (1629) | 0.12 (17) | 1627 | -0.04 (-0.17, 0.10) | -- |
| Acute Respiratory Infection (ARI) (proportion cases) | -- | -- | 4620 | -- | .51 |
| *Pop. Dens. Tertile 1* | 0.20 (1157) | 0.18 (234) | 1382 | -0.04 (-0.11, 0.03) | -- |
| *Pop. Dens. Tertile 2* | 0.25 (1488) | 0.15 (137) | 1609 | -0.13 (-0.21, -0.04)* | -- |
| *Pop. Dens. Tertile 3* | 0.24 (1629) | 0.29 (17) | 1629 | 0.17 (-0.23, 0.57) | -- |
| **Subgroup p-value <.05; ** Interaction p-value <.20* | | | | | |

# **Table S12:** Analysis of effect modification by population density on community hygienic latrine coverage (the proportion of compounds within range with only hygienic latrines) within 50 m of study compounds. Exposure modeled as a continuous variable in 10 percentage point increments. All models are adjusted and include robust standard errors.

|  | *n* | Adjusted association between exposure and outcome within subgroup of population density | Interaction term p-value (continuous form of population density) |
| --- | --- | --- | --- |
| ***Log10 E. coli MPN Differences*** | -- | -- | -- |
| Stored Water | 2123 | -- | .11** |
| *Pop. Dens. Tertile 1* | 560 | 0.01 (-0.01, 0.03) | -- |
| *Pop. Dens. Tertile 2* | 869 | -0.02 (-0.05, 0.00) | -- |
| *Pop. Dens. Tertile 3* | 694 | -0.05 (-0.09, -0.01)* | -- |
| Child Hand Rinses | 2443 | -- | .79 |
| *Pop. Dens. Tertile 1* | 634 | 0.00 (-0.02, 0.03) | -- |
| *Pop. Dens. Tertile 2* | 998 | -0.01 (-0.03, 0.02) | -- |
| *Pop. Dens. Tertile 3* | 811 | -0.01 (-0.05, 0.03) | -- |
| Caregiver Hand Rinses | 2486 | -- | .07** |
| *Pop. Dens. Tertile 1* | 654 | 0.02 (0.00, 0.04) | -- |
| *Pop. Dens. Tertile 2* | 1015 | 0.02 (-0.01, 0.04) | -- |
| *Pop. Dens. Tertile 3* | 817 | -0.04 (-0.08, 0.00) | -- |
| ***Prevalence Ratios*** | -- | -- | -- |
| Diarrheal Disease | 4419 | -- | .01** |
| *Pop. Dens. Tertile 1* | 1040 | 1.04 (1.00, 1.08)* | -- |
| *Pop. Dens. Tertile 2* | 1894 | 0.98 (0.94, 1.03) | -- |
| *Pop. Dens. Tertile 3* | 1485 | 0.95 (0.88, 1.02) | -- |
| Acute Respiratory Infection (ARI) | 4422 | -- | .27 |
| *Pop. Dens. Tertile 1* | 1040 | 1.00 (0.96, 1.04) | -- |
| *Pop. Dens. Tertile 2* | 1898 | 1.00 (0.96, 1.04) | -- |
| *Pop. Dens. Tertile 3* | 1484 | 1.03 (0.97, 1.10) | -- |
| ***Prevalence Differences*** | -- | -- | -- |
| Diarrheal Disease | 4419 | -- | .01** |
| *Pop. Dens. Tertile 1* | 1040 | 0.00 (0.00, 0.01) | -- |
| *Pop. Dens. Tertile 2* | 1894 | 0.00 (-0.01, 0.00) | -- |
| *Pop. Dens. Tertile 3* | 1485 | -0.01 (-0.02, 0.00) | -- |
| Acute Respiratory Infection (ARI) | 4422 | -- | .27 |
| *Pop. Dens. Tertile 1* | 1040 | 0.00 (-0.01, 0.01) | -- |
| *Pop. Dens. Tertile 2* | 1898 | 0.00 (-0.01, 0.01) | -- |
| *Pop. Dens. Tertile 3* | 1484 | 0.01 (-0.01, 0.02) | -- |
| **Subgroup p-value <.05; ** Interaction p-value <.20* | | | |

# **Table S13:** Analysis of effect modification by population density on community hygienic latrine coverage (the proportion of compounds within range with only hygienic latrines) within 100 m of study compounds. Exposure modeled as a continuous variable in 10 percentage point increments. All models are adjusted and include robust standard errors.

|  | *n* | Adjusted association between exposure and outcome within subgroup of population density | Interaction term p-value (continuous form of population density) |
| --- | --- | --- | --- |
| ***Log10 E. coli MPN Differences*** | -- | -- | -- |
| Stored Water | 2214 | -- | .75 |
| *Pop. Dens. Tertile 1* | 725 | -0.02 (-0.05, 0.02) | -- |
| *Pop. Dens. Tertile 2* | 775 | -0.02 (-0.05, 0.02) | -- |
| *Pop. Dens. Tertile 3* | 714 | -0.06 (-0.11, -0.02)* | -- |
| Child Hand Rinses | 2550 | -- | .74 |
| *Pop. Dens. Tertile 1* | 829 | 0.00 (-0.03, 0.03) | -- |
| *Pop. Dens. Tertile 2* | 857 | -0.03 (-0.06, 0.01) | -- |
| *Pop. Dens. Tertile 3* | 864 | -0.04 (-0.08, 0.01) | -- |
| Caregiver Hand Rinses | 2594 | -- | .17** |
| *Pop. Dens. Tertile 1* | 848 | 0.00 (-0.03, 0.02) | -- |
| *Pop. Dens. Tertile 2* | 871 | 0.01 (-0.03, 0.05) | -- |
| *Pop. Dens. Tertile 3* | 875 | -0.02 (-0.08, 0.03) | -- |
| ***Prevalence Ratios*** | -- | -- | -- |
| Diarrheal Disease | 4617 | -- | <.01** |
| *Pop. Dens. Tertile 1* | 1382 | 1.05 (0.98, 1.11) | -- |
| *Pop. Dens. Tertile 2* | 1608 | 0.99 (0.93, 1.06) | -- |
| *Pop. Dens. Tertile 3* | 1627 | 0.95 (0.87, 1.04) | -- |
| Acute Respiratory Infection (ARI) | 4620 | -- | .54 |
| *Pop. Dens. Tertile 1* | 1382 | 0.99 (0.94, 1.04) | -- |
| *Pop. Dens. Tertile 2* | 1609 | 1.01 (0.95, 1.08) | -- |
| *Pop. Dens. Tertile 3* | 1629 | 1.02 (0.97, 1.08) | -- |
| ***Prevalence Differences*** | -- | -- | -- |
| Diarrheal Disease | 4617 | -- | <.01** |
| *Pop. Dens. Tertile 1* | 1382 | 0.01 (0.00, 0.01) | -- |
| *Pop. Dens. Tertile 2* | 1608 | 0.00 (-0.01, 0.01) | -- |
| *Pop. Dens. Tertile 3* | 1627 | -0.01 (-0.02, 0.01) | -- |
| Acute Respiratory Infection (ARI) | 4620 | -- | .48 |
| *Pop. Dens. Tertile 1* | 1382 | 0.00 (-0.01, 0.01) | -- |
| *Pop. Dens. Tertile 2* | 1609 | 0.00 (-0.01, 0.02) | -- |
| *Pop. Dens. Tertile 3* | 1629 | 0.00 (-0.01, 0.02) | -- |
| **Subgroup p-value <.05; ** Interaction p-value <.20* | | | |

# **Table S14:** Analysis of effect modification by season (monsoon vs. dry) on community any latrine coverage (the proportion of compounds within range with at least one latrine) within 50 m of study compounds. Exposure modeled as a binary variable (100% coverage vs. <100%). All models are adjusted and include robust standard errors.

|  | <100% of Compounds within 50 m Have At Least One Latrine | 100% of Compounds within 50 m Have At Least One Latrine | Adjusted association between exposure and outcome within subgroup of population density | | Interaction Term p-Value (continuous form of population density) |
| --- | --- | --- | --- | --- | --- |
|  | Mean (n) or Proportion (n) of Outcome | | *n* | Estimate (95% CI) |  |
| ***Log10 E. coli MPN Differences*** | -- | -- | -- | -- | -- |
| Stored Water (mean) | -- | -- | 2129 | -- | .46 |
| *Monsoon Season* | 0.95 (256) | 0.77 (816) | 1096 | -0.18 (-0.33, -0.03)* | -- |
| *Dry Season* | 1.39 (276) | 1.18 (862) | 1033 | -0.12 (-0.27, 0.03) | -- |
| Child Hand Rinses (mean) | -- | -- | 2450 | -- | .55 |
| *Monsoon Season* | 1.51 (283) | 1.37 (943) | 1247 | -0.14 (-0.28, 0.00)* | -- |
| *Dry Season* | 1.75 (299) | 1.54 (970) | 1203 | -0.11 (-0.26, 0.03) | -- |
| Caregiver Hand Rinses (mean) | -- | -- | 2493 | -- | .53 |
| *Monsoon Season* | 1.64 (287) | 1.41 (958) | 1265 | -0.14 (-0.28, -0.01)* | -- |
| *Dry Season* | 1.63 (305) | 1.44 (979) | 1228 | -0.18 (-0.36, -0.01)* | -- |
| ***Prevalence Ratios*** | -- | -- | -- | -- | -- |
| Diarrheal Disease (proportion cases) | -- | -- | 4426 | -- | .59 |
| *Monsoon Season* | 0.15 (403) | 0.13 (1371) | 2670 | 0.80 (0.61, 1.05) | -- |
| *Dry Season* | 0.18 (700) | 0.15 (1997) | 1756 | 0.88 (0.66, 1.17) | -- |
| Acute Respiratory Infection (ARI) (proportion cases) | -- | -- | 4429 | -- | .83 |
| *Monsoon Season* | 0.32 (403) | 0.26 (1371) | 2670 | 0.85 (0.68, 1.06) | -- |
| *Dry Season* | 0.24 (700) | 0.19 (1997) | 1759 | 0.83 (0.66, 1.04) | -- |
| ***Prevalence Differences*** | -- | -- | -- | -- | -- |
| Diarrheal Disease (proportion cases) | -- | -- | 4426 | -- | .48 |
| *Monsoon Season* | 0.15 (403) | 0.13 (1371) | 2670 | -0.03 (-0.08, 0.01) | -- |
| *Dry Season* | 0.18 (700) | 0.15 (1997) | 1756 | -0.01 (-0.06, 0.03) | -- |
| Acute Respiratory Infection (ARI) (proportion cases) | -- | -- | 4429 | -- | .83 |
| *Monsoon Season* | 0.32 (403) | 0.26 (1371) | 2670 | -0.04 (-0.09, 0.01) | -- |
| *Dry Season* | 0.24 (700) | 0.19 (1997) | 1759 | -0.05 (-0.12, 0.01) | -- |
| **Subgroup p-value <.05; ** Interaction p-value <.20* | | | | | |

# **Table S15**: Analysis of effect modification by season (monsoon vs. dry) on community hygienic latrine coverage (the proportion of compounds within range with only hygienic latrines) within 50 m of study compounds. Exposure modeled as a binary variable (100% coverage vs. <100%). All models are adjusted and include robust standard errors.

|  | <100% of Compounds within 50 m Have Only Hygienic Latrines | 100% of Compounds within 50 m Have Only Hygienic Latrines | Adjusted association between exposure and outcome within subgroup of population density | | Interaction Term p-Value (continuous form of population density) |
| --- | --- | --- | --- | --- | --- |
|  | Mean (n) or Proportion (n) of Outcome | | *n* | Estimate (95% CI) |  |
| ***Log10 E. coli MPN Differences*** | -- | -- | -- | -- | -- |
| Stored Water (mean) | -- | -- | 2123 | -- | .86 |
| *Monsoon Season* | 0.82 (853) | 0.78 (215) | 1094 | 0.01 (-0.16, 0.18) | -- |
| *Dry Season* | 1.24 (881) | 1.21 (255) | 1029 | 0.03 (-0.14, 0.20) | -- |
| Child Hand Rinses (mean) | -- | -- | 2443 | -- | .01** |
| *Monsoon Season* | 1.41 (969) | 1.40 (253) | 1244 | -0.14 (-0.27, 0.00)* | -- |
| *Dry Season* | 1.63 (983) | 1.44 (283) | 1199 | 0.06 (-0.09, 0.20) | -- |
| Caregiver Hand Rinses (mean) | -- | -- | 2486 | -- | .01** |
| *Monsoon Season* | 1.49 (980) | 1.39 (261) | 1262 | 0.16 (0.02, 0.30)* | -- |
| *Dry Season* | 1.45 (993) | 1.59 (288) | 1224 | -0.04 (-0.19, 0.11) | -- |
| ***Prevalence Ratios*** | -- | -- | -- | -- | -- |
| Diarrheal Disease (proportion cases) | -- | -- | 4419 | -- | .07** |
| *Monsoon Season* | 0.14 (1371) | 0.12 (400) | 2666 | 1.32 (0.96, 1.81) | -- |
| *Dry Season* | 0.14 (2150) | 0.19 (543) | 1753 | 0.88 (0.65, 1.19) | -- |
| Acute Respiratory Infection (ARI) (proportion cases) | -- | -- | 4422 | -- | .25 |
| *Monsoon Season* | 0.27 (1371) | 0.26 (400) | 2666 | 0.86 (0.68, 1.07) | -- |
| *Dry Season* | 0.21 (2150) | 0.17 (543) | 1756 | 0.96 (0.77, 1.19) | -- |
| ***Prevalence Differences*** | -- | -- | -- | -- | -- |
| Diarrheal Disease (proportion cases) | -- | -- | 4419 | -- | .07** |
| *Monsoon Season* | 0.14 (1371) | 0.12 (400) | 2666 | 0.05 (-0.01, 0.11) | -- |
| *Dry Season* | 0.14 (2150) | 0.19 (543) | 1753 | -0.01 (-0.05, 0.03) | -- |
| Acute Respiratory Infection (ARI) (proportion cases) | -- | -- | 4422 | -- | .34 |
| *Monsoon Season* | 0.27 (1371) | 0.26 (400) | 2666 | -0.03 (-0.07, 0.01) | -- |
| *Dry Season* | 0.21 (2150) | 0.17 (543) | 1756 | -0.01 (-0.07, 0.04) | -- |
| **Subgroup p-value <.05; ** Interaction p-value <.20* | | | | | |

# **Figure S2:** Effect modification by season (monsoon vs. dry) on community-level any latrine coverage (the proportion of compounds within range with at least one latrine) and community hygienic latrine coverage (the proportion of compounds within range with only hygienic latrines) within 100 m of study compounds and study outcomes. Exposures were modeled as binary variables (100% vs. <100% coverage). Plots show subgroup estimates within each season. P-values are for the interaction term between season and the exposure. All models are adjusted and include robust standard errors.

# **Table S16:** Analysis of effect modification by season (monsoon vs. dry) on community any latrine coverage (the proportion of compounds within range with at least one latrine) within 100 m of study compounds. Exposure modeled as a binary variable (100% coverage vs. <100%). All models are adjusted and include robust standard errors.

|  | <100% of Compounds within 100 m Have At Least One Latrine | 100% of Compounds within 100 m Have At Least One Latrine | Adjusted association between exposure and outcome within subgroup of population density | | Interaction Term p-Value (continuous form of population density) |
| --- | --- | --- | --- | --- | --- |
|  | Mean (n) or Proportion (n) of Outcome | | *n* | Estimate (95% CI) |  |
| ***Log10 E. coli MPN Differences*** | -- | -- | -- | -- | -- |
| Stored Water (mean) | -- | -- | 2221 | -- | .61 |
| *Monsoon Season* | 0.88 (477) | 0.75 (648) | 1136 | -0.02 (-0.15, 0.10) | -- |
| *Dry Season* | 1.29 (496) | 1.19 (685) | 1085 | -0.07 (-0.20, 0.05) | -- |
| Child Hand Rinses (mean) | -- | -- | 2556 | -- | .60 |
| *Monsoon Season* | 1.47 (544) | 1.36 (740) | 1296 | -0.07 (-0.19, 0.05) | -- |
| *Dry Season* | 1.67 (547) | 1.54 (774) | 1260 | -0.05 (-0.16, 0.07) | -- |
| Caregiver Hand Rinses (mean) | -- | -- | 2601 | -- | .31 |
| *Monsoon Season* | 1.58 (553) | 1.39 (752) | 1314 | -0.06 (-0.19, 0.06) | -- |
| *Dry Season* | 1.56 (556) | 1.44 (779) | 1287 | -0.14 (-0.28, 0.00) | -- |
| ***Prevalence Ratios*** | -- | -- | -- | -- | -- |
| Diarrheal Disease (proportion cases) | -- | -- | 4627 | -- | .59 |
| *Monsoon Season* | 0.15 (772) | 0.12 (1096) | 2777 | 0.84 (0.66, 1.07) | -- |
| *Dry Season* | 0.17 (1249) | 0.14 (1555) | 1850 | 0.79 (0.62, 1.02) | -- |
| Acute Respiratory Infection (ARI) (proportion cases) | -- | -- | 4630 | -- | .82 |
| *Monsoon Season* | 0.28 (772) | 0.26 (1096) | 2777 | 0.91 (0.75, 1.11) | -- |
| *Dry Season* | 0.22 (1249) | 0.19 (1555) | 1853 | 0.93 (0.76, 1.14) | -- |
| ***Prevalence Differences*** | -- | -- | -- | -- | -- |
| Diarrheal Disease (proportion cases) | -- | -- | 4627 | -- | .74 |
| *Monsoon Season* | 0.15 (772) | 0.12 (1096) | 2777 | -0.02 (-0.06, 0.01) | -- |
| *Dry Season* | 0.17 (1249) | 0.14 (1555) | 1850 | -0.03 (-0.06, 0.01) | -- |
| Acute Respiratory Infection (ARI) (proportion cases) | -- | -- | 4630 | -- | .99 |
| *Monsoon Season* | 0.28 (772) | 0.26 (1096) | 2777 | -0.02 (-0.06, 0.02) | -- |
| *Dry Season* | 0.22 (1249) | 0.19 (1555) | 1853 | -0.02 (-0.07, 0.03) | -- |
| **Subgroup p-value <.05; ** Interaction p-value <.20* | | | | | |

# **Table S17**: Analysis of effect modification by season (monsoon vs. dry) on community hygienic latrine coverage (the proportion of compounds within range with only hygienic latrines) within 100 m of study compounds. Exposure modeled as a binary variable (100% coverage vs. <100%). All models are adjusted and include robust standard errors.

|  | <100% of Compounds within 100 m Have Only Hygienic Latrines | 100% of Compounds within 100 m Have Only Hygienic Latrines | Adjusted association between exposure and outcome within subgroup of population density | | Interaction Term p-Value (continuous form of population density) |
| --- | --- | --- | --- | --- | --- |
|  | Mean (n) or Proportion (n) of Outcome | | *n* | Estimate (95% CI) |  |
| ***Log10 E. coli MPN Differences*** | -- | -- | -- | -- | -- |
| Stored Water (mean) | -- | -- | 2214 | -- | .64 |
| *Monsoon Season* | 0.82 (1017) | 0.67 (104) | 1133 | -0.10 (-0.36, 0.15) | -- |
| *Dry Season* | 1.26 (1056) | 1.06 (122) | 1081 | 0.01 (-0.21, 0.22) | -- |
| Child Hand Rinses (mean) | -- | -- | 2550 | -- | .32 |
| *Monsoon Season* | 1.41 (1163) | 1.34 (117) | 1294 | -0.03 (-0.22, 0.16) | -- |
| *Dry Season* | 1.61 (1185) | 1.50 (133) | 1256 | 0.01 (-0.17, 0.18) | -- |
| Caregiver Hand Rinses (mean) | -- | -- | 2594 | -- | .12** |
| *Monsoon Season* | 1.49 (1181) | 1.33 (120) | 1311 | 0.10 (-0.12, 0.33) | -- |
| *Dry Season* | 1.49 (1198) | 1.51 (134) | 1283 | -0.05 (-0.25, 0.16) | -- |
| ***Prevalence Ratios*** | -- | -- | -- | -- | -- |
| Diarrheal Disease (proportion cases) | -- | -- | 4617 | -- | .63 |
| *Monsoon Season* | 0.13 (1706) | 0.14 (158) | 2771 | 1.13 (0.73, 1.73) | -- |
| *Dry Season* | 0.15 (2568) | 0.18 (230) | 1846 | 1.04 (0.65, 1.65) | -- |
| Acute Respiratory Infection (ARI) (proportion cases) | -- | -- | 4620 | -- | .36 |
| *Monsoon Season* | 0.27 (1706) | 0.23 (158) | 2771 | 0.72 (0.45, 1.16) | -- |
| *Dry Season* | 0.21 (2568) | 0.14 (230) | 1849 | 0.85 (0.60, 1.19) | -- |
| ***Prevalence Differences*** | -- | -- | -- | -- | -- |
| Diarrheal Disease (proportion cases) | -- | -- | 4617 | -- | .61 |
| *Monsoon Season* | 0.13 (1706) | 0.14 (158) | 2771 | 0.03 (-0.06, 0.11) | -- |
| *Dry Season* | 0.15 (2568) | 0.18 (230) | 1846 | 0.01 (-0.06, 0.09) | -- |
| Acute Respiratory Infection (ARI) (proportion cases) | -- | -- | 4620 | -- | .50 |
| *Monsoon Season* | 0.27 (1706) | 0.23 (158) | 2771 | -0.06 (-0.13, 0.01) | -- |
| *Dry Season* | 0.21 (2568) | 0.14 (230) | 1849 | -0.04 (-0.12, 0.04) | -- |
| **Subgroup p-value <.05; ** Interaction p-value <.20* | | | | | |

# **Table S18:** Analysis of effect modification by season (monsoon vs. dry) on community hygienic latrine coverage (the proportion of compounds within range with only hygienic latrines) within 50 m of study compounds. Exposure modeled as a continuous variable in 10 percentage point increments. All models are adjusted and include robust standard errors.

|  | *n* | Adjusted association between exposure and outcome within subgroup of population density | Interaction term p-value (continuous form of population density) |
| --- | --- | --- | --- |
| ***Log10 E. coli MPN Differences*** | -- | -- | -- |
| Stored Water | 2123 | -- | .49 |
| *Monsoon Season* | 1094 | -0.01 (-0.03, 0.01) | -- |
| *Dry Season* | 1029 | -0.02 (-0.03, 0.00) | -- |
| Child Hand Rinses | 2443 | -- | .03** |
| *Monsoon Season* | 1244 | -0.01 (-0.03, 0.01) | -- |
| *Dry Season* | 1199 | 0.01 (-0.01, 0.03) | -- |
| Caregiver Hand Rinses | 2486 | -- | .09** |
| *Monsoon Season* | 1262 | 0.02 (0.00, 0.04) | -- |
| *Dry Season* | 1224 | 0.00 (-0.02, 0.02) | -- |
| ***Prevalence Ratios*** | -- | -- | -- |
| Diarrheal Disease | 4419 | -- | .19** |
| *Monsoon Season* | 2666 | 1.01 (0.97, 1.05) | -- |
| *Dry Season* | 1753 | 0.98 (0.94, 1.02) | -- |
| Acute Respiratory Infection (ARI) | 4422 | -- | .32 |
| *Monsoon Season* | 2666 | 0.99 (0.96, 1.02) | -- |
| *Dry Season* | 1756 | 1.00 (0.97, 1.03) | -- |
| ***Prevalence Differences*** | -- | -- | -- |
| Diarrheal Disease | 4419 | -- | .23 |
| *Monsoon Season* | 2666 | 0.00 (0.00, 0.01) | -- |
| *Dry Season* | 1753 | 0.00 (-0.01, 0.00) | -- |
| Acute Respiratory Infection (ARI) | 4422 | -- | .40 |
| *Monsoon Season* | 2666 | 0.00 (-0.01, 0.00) | -- |
| *Dry Season* | 1756 | 0.00 (-0.01, 0.01) | -- |
| **Subgroup p-value <.05; ** Interaction p-value <.20* | | | |

# **Table S19:** Analysis of effect modification by season (monsoon vs. dry) on community hygienic latrine coverage (the proportion of compounds within range with only hygienic latrines) within 100 m of study compounds. Exposure modeled as a continuous variable in 10 percentage point increments. All models are adjusted and include robust standard errors.

|  | *n* | Adjusted association between exposure and outcome within subgroup of population density | Interaction term p-value (continuous form of population density) |
| --- | --- | --- | --- |
| ***Log10 E. coli MPN Differences*** | -- | -- | -- |
| Stored Water | 2214 | -- | .46 |
| *Monsoon Season* | 1133 | -0.01 (-0.04, 0.01) | -- |
| *Dry Season* | 1081 | -0.03 (-0.05, 0.00)* | -- |
| Child Hand Rinses | 2550 | -- | .88 |
| *Monsoon Season* | 1294 | -0.01 (-0.03, 0.02) | -- |
| *Dry Season* | 1256 | -0.01 (-0.04, 0.01) | -- |
| Caregiver Hand Rinses | 2594 | -- | .03** |
| *Monsoon Season* | 1311 | 0.01 (-0.02, 0.04) | -- |
| *Dry Season* | 1283 | -0.02 (-0.04, 0.01) | -- |
| ***Prevalence Ratios*** | -- | -- | -- |
| Diarrheal Disease | 4617 | -- | .30 |
| *Monsoon Season* | 2771 | 1.01 (0.96, 1.07) | -- |
| *Dry Season* | 1846 | 0.99 (0.94, 1.04) | -- |
| Acute Respiratory Infection (ARI) | 4620 | -- | .52 |
| *Monsoon Season* | 2771 | 1.00 (0.96, 1.04) | -- |
| *Dry Season* | 1849 | 1.01 (0.97, 1.05) | -- |
| ***Prevalence Differences*** | -- | -- | -- |
| Diarrheal Disease | 4617 | -- | .34 |
| *Monsoon Season* | 2771 | 0.00 (-0.01, 0.01) | -- |
| *Dry Season* | 1846 | 0.00 (-0.01, 0.01) | -- |
| Acute Respiratory Infection (ARI) | 4620 | -- | .57 |
| *Monsoon Season* | 2771 | 0.00 (-0.01, 0.01) | -- |
| *Dry Season* | 1849 | 0.00 (-0.01, 0.01) | -- |
| **Subgroup p-value <.05; ** Interaction p-value <.20* | | | |
